# Early disruptions in brain capillary blood flow control in a mouse model of Alzheimer’s disease

**DOI:** 10.1085/jgp.202614014

**Published:** 2026-10-15

**Authors:** Ashwini Hariharan, Abigail Vigderman, Dominic P. Isaacs, Nick Weir, Gyubyung Park, Serena Edwards, Thomas A. Longden

**Affiliations:** 1 Department of Pharmacology & Physiology, University of Maryland School of Medicine, Baltimore, MD, USA

## Abstract

In Alzheimer’s disease (AD), vascular dysfunction and impaired control of brain blood flow can disrupt cellular bioenergetics decades before cognitive decline. Identifying the earliest vascular changes is therefore critical for developing therapies that intervene before the cascading events leading to neuronal decline. Brain energy supply is dynamically controlled by the cerebral vasculature, where capillary thin-strand pericytes are emerging as key players. We recently identified a central role for ATP-sensitive potassium (K^+^, K_ATP_) channels in enabling thin-strand pericytes to sense local neuronal activity and generate hyperpolarizing signals that are transmitted via capillary endothelial cells to upstream arterioles, eliciting increases in blood flow. Importantly, thin-strand pericyte K_ATP_ channels are the centerpiece of an electro-metabolic “energy switch” mechanism that couples decreases in local glucose to hyperemia, providing a failsafe to ensure continual energy substrate supply to support neuronal function. Disruption of this mechanism could therefore precipitate an imbalance between demand and supply, creating an energy gap for neurons that leads to deleterious consequences. We demonstrate that capillary control of blood flow is disrupted in the early stages of disease progression in mice expressing familial AD mutations (5xFAD). Transgenic 5xFAD mice exhibit early disruption of capillary signaling, beginning with dysfunctional endothelial inward rectifier K^+^ (K_ir_)2.1 channels at 2–4 mo, disabling transmission of K_ATP_-mediated signals to upstream blood flow control machinery, followed by direct impairment of pericyte K_ATP_ channel function by 6–8 mo. Together, these deficits cause early and sustained failure of electrical signaling from deep capillary pericytes to upstream arterioles and arteriole–capillary transition (ACT) zone vessels, limiting K_ATP_-dependent energy-switch-mediated blood flow control. Our findings define a temporal and mechanistic framework linking familial AD mutations to early capillary signaling dysfunction and highlight preservation of capillary blood flow control as a potential therapeutic target to protect against downstream effects of amyloid β accumulation.

## Teaser

Hariharan et al. show that in a mouse model of Alzheimer’s disease, capillary blood flow control fails early: endothelial K_ir_2.1 currents decline at 2–4 mo, followed by pericyte K_ATP_ channel impairment by 6–8 mo. Notably, the early K_ir_2.1 deficit can be rescued by restoring PIP_2_ levels, which reinstates communication between deep capillary pericytes and upstream arterioles.

## Introduction

Disruption of the mechanisms governing the metabolic supply-and-demand relationship that is integral to brain energy homeostasis can have deleterious consequences for brain health and is a major contributor to the development of age-related disorders such as Alzheimer’s disease (AD). The steady increase in global life expectancy and the higher prevalence of AD among the aging population make it one of the most common disorders to affect the elderly and the third-leading cause of death in people over the age of 75 years (Shreya et al., 2025). Importantly, mid-life vascular dysfunction can independently affect amyloid deposition (Gottesman et al., 2017) and is associated with cognitive decline later in life (Debette et al., 2011; Guan et al., 2025; Knopman et al., 2018), suggesting a causative link between aging, brain blood flow control, and development of AD. Indeed, emerging research suggests that the underlying pathological features of AD begin decades before the symptoms of cognitive decline emerge (Barthélemy et al., 2020; Braak et al., 2011; Jack et al., 2009) and vascular dysfunction appears to be one of the earliest detectable anomalies and may even be the inciting pathology (Iturria-Medina et al., 2016).

The elaborate architecture and sophisticated molecular organization of the cerebrovasculature ensure that mediators of neuronal activity are detected rapidly, and in return, the key energy substrates, glucose and oxygen, are efficiently delivered via increases in local blood flow through the mechanisms of neurovascular coupling. The resulting functional hyperemia serves a range of homeostatic purposes beyond energy delivery by also regulating temperature, maintaining pH, and clearing waste products (Drew, 2022). Accordingly, blood flow control is essential for brain health, and its disruption could precipitate progressive neuronal decline and cell death.

At the brain surface, pial arteries dive orthogonally into the parenchyma to form penetrating arterioles (PAs), which then branch into a dense network of bifurcating capillaries that form the vast majority of the cerebrovascular network (Cipolla, 2009). Various types of mural cells lend different contractile properties to these tiers of blood vessels. Pial arteries and PAs are covered by smooth muscle cells (SMCs) that are coupled to the underlying endothelium via myo-endothelial gap junctions. As the capillaries branch from the PA, SMCs transition into pericytes that have distinct molecular and physiological characteristics according to the branch order that they reside upon (Grant et al., 2019; Hariharan et al., 2020; Hartmann et al., 2022; He et al., 2018; Vanlandewijck et al., 2018). The initial branches (approximately first to fourth order) of the capillaries, hereafter referred to as arteriole–capillary transition (ACT) zone capillaries (Bonney et al., 2024), are covered by ensheathing pericytes that can rapidly contract and relax to adjust the diameter of the underlying vessel (Hartmann et al., 2021). Deeper in the capillary bed (equal to or higher than the fifth order), the long processes of thin-strand pericytes cover the capillary wall and exert subtle contractile forces to control underlying capillary diameter over slower time-scales than their ensheathing contractile counterparts (Berthiaume et al., 2018; Hartmann et al., 2021). Like SMCs, pericytes are also intimately connected to the underlying capillary endothelial cells (cECs) via gap junctions made up of an array of connexin isoforms (Kovacs-Oller et al., 2020; Krolak et al., 2025). Together with endothelial tight junctions, this coupling establishes a vascular syncytium that enables rapid electrical communication along the capillary-arteriolar axis to direct robust increases in blood flow in a spatially precise manner (Longden et al., 2017; Longden et al., 2023).

A growing interest in the physiological roles of pericytes has revealed diverse mechanisms by which they control blood flow in the brain (Hariharan et al., 2022; Hartmann et al., 2021; Isaacs et al., 2024; Phillips et al., 2023; Rungta et al., 2018; Sancho et al., 2022; Xiang et al., 2026) and the contributions of their disruption to disease processes (Bowen et al., 2025; Hirunpattarasilp et al., 2019; Korte et al., 2024; Nortley et al., 2019). Pericytes are optimally positioned to monitor local neuronal energy needs and express a broad repertoire of ion channels and receptors (Hariharan et al., 2020), enabling them to participate in electro-metabolic control of brain blood flow (Longden and Lederer, 2024). Indeed, we identified that expression of the vascular isoform of the ATP-sensitive potassium (K^+^; K_ATP_) channel imbues thin-strand pericytes with the ability to generate hyperpolarizing signals that can be transmitted to the underlying cEC to generate retrograde electrical signaling via inward-rectifier potassium (K_ir_)2.1 channels (Longden et al., 2017), causing an increase in the diameter of the connected, upstream PA and evoking an elevation of blood flow (Hariharan et al., 2022; Isaacs et al., 2024). These hyperpolarizing signals, in turn, are counterbalanced by the thin-strand pericyte calcium (Ca^2+^)-activated chloride (Cl^−^) channels, which oppose hyperpolarizing signals and tether the membrane potential to its resting level (Xiang et al., 2026). This organization led us to propose the concept of the capillary computational unit (CCU), in which pericytes operate as multimodal sensors continually surveying neuronal energy needs and generating electrical output, which is then fed into and reliant on cECs, which are optimized for long-distance signal transmission (Longden, 2026).

A key controller of electrical output of thin-strand pericytes appears to be the level of local glucose, subtle decreases in which activate thin-strand pericyte K_ATP_ channels to drive blood flow elevations (Hariharan et al., 2022). We previously showed that this energy switch mechanism enables thin-strand pericytes to optimize substrate delivery, providing a failsafe against substrate deficits and protecting ongoing neuronal health and function. Any disruption of this mechanism of brain energy homeostasis could therefore lead to deleterious consequences for neuronal function and long-term brain health.

Transgenic (Tg) mouse models harboring amyloid beta (Aβ) or tau pathology are commonly used to study the molecular mechanisms of cognitive decline in AD. The 5xFAD Tg mouse line overexpresses five mutations associated with familial AD; three of these (the Swedish [K670N, M671L], Florida [I716V], and London [V717I] mutations) occur in the gene encoding human amyloid precursor protein (*APP*), and two are found in human presenilin-1 (*PSEN1*; M146L and L286V) (Oakley et al., 2006). The combined expression of these mutations results in an overproduction of toxic Aβ_42_ peptides and accelerated amyloid plaque formation, making this a widely used model to study the downstream effects of amyloid pathology. In 5xFAD mice, elevated Aβ_42_ peptide levels are detected as early as 1.5 mo of age (Oakley et al., 2006). Amyloid deposits are notable in the hippocampal formation at 2 mo and in the cortex by 4–6 mo (Bader et al., 2023; Gail Canter et al., 2019; Oakley et al., 2006; Oblak et al., 2021), with behavioral impairments emerging by 5 mo of age (Forner et al., 2021; Oakley et al., 2006; Richard et al., 2015; Tang et al., 2016). Studies on cerebrovascular function in these mice reveal the presence of cerebral amyloid angiopathy (Giannoni et al., 2016) and hippocampal blood flow deficits as early as 3 mo (Lowerison et al., 2024), elevated capillary stalls occurring at 5–6 mo (Cruz Hernández et al., 2019), and reduced neurovascular coupling at 12–13 mo (Mughal et al., 2021). Despite the extensive use of the 5xFAD mouse line in AD research, it is not known how amyloidosis affects thin-strand pericyte K_ATP_ activity and its control over regional blood flow and neurovascular coupling. Here, we use a combination of single-cell electrophysiology and *in vivo* imaging to investigate the time course of changes in thin-strand pericyte K_ATP_ function and the energy switch mechanism in Tg 5xFAD mice and wild-type (Wt) littermates, focusing primarily on two stages of disease progression which we term “early” (2–4 mo) and “moderate” (6–8 mo). We demonstrate that in 5xFAD mice, cEC K_ir_2.1 activity breaks down at 2–4 mo, which collapses CCU function, and that pericyte K_ATP_-mediated hyperpolarization is directly disrupted later at 6–8 mo. The inability of CCU-generated signals to reach PAs and evoke hyperemia in young 5xFAD mice results in early and long-lasting deficits in thin-strand pericyte K_ATP_-mediated blood flow control, which may together severely impact neuronal health and function if left unchecked.

## Materials and methods

### Experimental animal model

This study used male and female Tg 5xFAD mice (Research Resource Identifier [RRID]:MMRRC_034840-JAX) and age-matched Wt littermate controls at ages of 2–4 mo (young adult), 6–8 mo (mature adult), and 10–15 mo (late middle-age). These ages were chosen based on previous studies showing the onset of amyloid accumulation in these mice at 2 mo and more severe disease pathology such as brain-wide plaque formation and cognitive impairments by 6–8 mo (Bader et al., 2023; Forner et al., 2021; Gail Canter et al., 2019; Oakley et al., 2006; Oblak et al., 2021; Richard et al., 2015; Tang et al., 2016). Mice were group-housed on a 12-h light:dark cycle with free access to food and water in the animal facility at the University of Maryland Baltimore. Genotyping was performed on tail samples collected from ∼P30–40 mice for the presence of target genes as recommended by Jackson Laboratories and detected by PCR. All animal care was conducted in accordance with the University of Maryland Institutional Animal Care and Use Committee (IACUC), and all animal experimental procedures described below received prior approval from IACUC and were conducted according to ARRIVE guidelines.

### AAV injections

AAV(BR1)-CAG-GFP (SL116035; SignaGen) was used to identify “bump-on-a-log” pericytes by exclusion as nonfluorescent cells on eGFP-positive capillaries for electrophysiology experiments (Xiang et al., 2026). For AAV transfection, mice were anesthetized with isoflurane (4%), and 100 μl of virus (1 × 10^12^ vg/ml) diluted in sterile saline was injected retro-orbitally. Mice were used for experiments after ∼4 wk to allow time for transfection.

### *In vivo* imaging of cerebral hemodynamics

Mice were anesthetized with isoflurane (4% induction, 1–2% maintenance, gradually reduced during surgery). 150 μl of fluorescein isothiocyanate (FITC)-dextran (10 mg/ml; FD2000S; Sigma-Aldrich) or tetramethylrhodamine isothiocyanate (TRITC)-dextran (40 mg/ml; T1287; Sigma-Aldrich) dissolved in sterile saline was injected retro-orbitally, followed by intraperitoneal (i.p.) injections of α-chloralose (50 mg/kg) and urethane (750 mg/kg). The skull was exposed with a midline incision, and a titanium headplate affixed with a mixture of dental cement and superglue was used to secure the head in a holding frame. A circular cranial window was drilled over the somatosensory cortex using a dental drill, and the dura mater was carefully removed to allow penetration of agents applied to the brain surface. Body temperature was maintained at 37°C using a rectal probe feedback-controlled electric heating pad. Pharmacological agents were dissolved in artificial cerebrospinal fluid (aCSF, composition: 124 mM NaCl, 3 mM KCl, 2 mM CaCl_2_, 2 mM MgCl_2_, 1.25 mM NaH_2_PO_4_, 26 mM NaHCO_3_, and 4 mM glucose) that was kept oxygenated (21% O_2_/5% CO_2_/balance N_2_) and warmed to 35–36°C. Images were acquired on a Scientifica Hyperscope (Scientifica) coupled to a Coherent Chameleon Vision II Titanium-Sapphire pulsed fs laser (Coherent). Baseline diameters of PAs and ACT zone capillaries were acquired using xyz stacks, and red blood cell (RBC) flux was measured by line scanning capillaries at 1 kHz. For superfusion experiments, baseline data were collected in circulating aCSF followed by application of drugs (10 μM pinacidil [15416; Cayman] or 1 μM BAY-876 [6199; Tocris Bioscience]). For pressure-ejection experiments on pericytes, TO-PRO-3 Iodide (1:25; T3605; Invitrogen) was applied to the brain surface for 20 min to allow the identification of pericytes. A pipette containing TRITC and 10 μM pinacidil or 10 mM K^+^ was maneuvered next to the pericyte of interest (for pinacidil) or next to a second- or third-order capillary (for 10 mM K^+^). The solution was pressure-ejected at 10–12 psi for 30 ms (pinacidil) or 5–10 ms (for 10 mM K^+^) to selectively apply the agent onto the target pericyte or capillary. In a subset of experiments, mice were systemically injected with the synthetic dipalmitoyl variant of phosphatidylinositol 4,5-bisphosphate (diC_16_-PIP_2_; 0.5 mg/kg, P-4516; Echelon Biosciences) ∼30 min before imaging.

### *In vivo* imaging of Aβ

Aβ was imaged *in vivo* using thioflavin S (Bacskai et al., 2001) in 12-mo-old Wt and Tg 5xFAD mice. Mice were prepared for *in vivo* imaging as described above, and retro-orbital injection of Texas red dextran (D1830; Invitrogen; 150 μl at 25 mg/ml in sterile saline) was used to visualize the vasculature. After removal of the dura, a solution of 0.01% thioflavin S in HEPES-buffered aCSF was applied to the brain surface for 20 min. Images of thioflavin S-positive Aβ and local vasculature were acquired at 750 nm on the Scientifica Hyperscope.

### Single-cell electrophysiology

Membrane potential responses of thin-strand pericytes to 10 μM pinacidil or 1 μM BAY-876 were measured using sharp microelectrode impalements (Hariharan et al., 2022), and cEC K_ir_2.1 currents were measured using conventional whole-cell patch-clamp electrophysiology (Longden et al., 2017). Mice were anesthetized with 4% isoflurane, rapidly decapitated, and brains were isolated into ice-cold enzyme solution (55 mM NaCl, 80 mM Na-glutamate, 5.6 mM KCl, 2 mM MgCl_2_, 10 mM HEPES, and 4 mM glucose, pH 7.3). Somatosensory cortex was dissected, and microvessels were isolated using Neural Tissue Dissociation Kit P (130-092-628; Miltenyi Biotec). First, tissue was minced into small pieces and incubated in enzyme P for 18 min at 37°C, followed by addition of enzyme A, homogenization (4–6 turns) with a 21-G needle, and incubation at 37°C for 8 min. Finally, the cell suspension was homogenized for 3–4 turns again, incubated at 37°C for 6 min, filtered through a 62-μm nylon mesh, and stored in ice-cold enzyme solution. For sharp microelectrode impalements, the recording chamber was perfused with bath solution (137 mM NaCl, 3 mM KCl, 2 mM CaCl_2_, 1 mM MgCl_2_, 10 mM HEPES, and 4 mM glucose, pH 7.4). Pericytes were identified as nonfluorescent cell bodies on eGFP-positive ECs and impaled with a sharp microelectrode (1 mm outer diameter, 0.58 mm inner diameter; Sutter Instruments, pulled to ∼100–200 MΩ) filled with 0.5 M KCl. Membrane potential was recorded at baseline, or in the presence of 10 μM pinacidil or 1 μM BAY-876 using Axoclamp 900A digital amplifier and a HS-2 headstage (Molecular Devices). Signals were digitized and stored using Axon Digidata 1550B and analyzed with Clampfit 10.7 software (Molecular Devices).

For measuring cEC K_ir_2.1 currents, borosilicate pipettes (1.5 mm outer diameter, 1.17 mm inner diameter; Sutter Instruments) were fire-polished to give a resistance of 5–6 MΩ and filled with internal solution containing 10 mM NaOH, 11.4 mM KOH, 128.6 mM KCl, 1.1 mM MgCl_2_, 3.2 mM CaCl_2_, 5 mM EGTA, and 10 mM HEPES (∼300 nM free Ca^2+^; pH 7.2). K_ir_2.1 currents were measured in bath solution containing 80 mM NaCl, 60 mM KCl, 1 mM MgCl_2_, 10 mM HEPES, 4 mM glucose, and 2 mM CaCl_2_ (pH 7.4). In a subset of experiments, the intracellular solution was supplemented with the synthetic dioctanoyl PIP_2_ variant diC_8_-PIP_2_ (10 μM, P-4508; Echelon Biosciences). Currents were amplified using a MultiClamp 700B amplifier (Molecular Devices), filtered at 1 kHz, digitized at 10 kHz using a Digidata 1440A (Molecular Devices), and analyzed with Clampfit 10.7 software (Molecular Devices).

### Immunohistochemistry

The expression of vascular glucose transporter (GLUT)1 in 6–8-mo-old Tg 5xFAD and age-matched Wt controls was quantified via immunohistochemistry. Mice were anesthetized with isoflurane and underwent cardiac perfusion with ice-cold PBS followed by 4% paraformaldehyde (PFA). Brains were extracted and stored in 4% PFA overnight at 4°C, then sectioned on a Leica VT1000 S vibratome into 100-µm slices on the following day and stored in PBS containing 0.005% sodium azide at 4°C until use. On day 1 of immunostaining, slices were washed three times for 10 min in 0.1% Triton X-100 in PBS (PBST), permeabilized in 0.3% PBST for 30 min at room temperature, and then blocked using 4% bovine serum albumin in 0.1% PBST for 60 min at room temperature. Slices were then incubated with a primary rabbit anti-SLC2A1 (GLUT1) polyclonal antibody (1:500, HPA031345; Atlas Antibodies, RRID:AB_2673835) and an isolectin GS-IB4 Alexa-488 conjugate (1:500, I21411; Invitrogen, RRID:AB_2314662) overnight at 4°C. The next day, slices were washed three times for 10 min in 0.1% PBST followed by incubation in secondary antibody (1:500, donkey anti-rabbit Alexa Fluor 647; A-31573; Invitrogen, RRID:AB_2536183) for 2 h at room temperature. Slices were again washed three times for 10 min in 0.1% PBST and mounted on slides. All incubations and washes were done on an orbital shaker, and no-primary antibody negative controls were included in every experiment. Slides were imaged on a Nikon AX confocal microscope.

### RT-qPCR of isolated vessels

mRNA of target genes was measured in vessels isolated from barrel cortices of 6–8-mo-old mice (Bjørnholm et al., 2023). Following euthanasia, brains were extracted and placed in ice-cold primary wash solution (phenol red-free Dulbecco’s modified Eagle medium [DMEM; 31053028; Invitrogen] with 1% penicillin/streptomycin [15140122; Invitrogen]). Barrel cortices from both hemispheres were dissected into a 1.5-ml tube containing 1 ml DMEM and cut into <1-mm^3^ pieces. Tissue was transferred to a 4 ml dissociation buffer (DMEM with 1 mg/ml collagenase, C6885; Sigma-Aldrich), triturated 20 times with a cut P1000 tip, and incubated at 37°C for 10 min with gentle shaking. Samples were triturated again 30 times with an intact P1000 tip and returned to 37°C for 12 min. The digest was filtered through a 70-μm strainer into a 50 ml tube; the strainer was rinsed with 10 ml primary wash medium, and cells were pelleted at 300 × *g* for 5 min at 4°C. The pellet was resuspended in 4.5 ml secondary wash medium (DMEM with 1% P/S and 0.5 mg/ml heparin, Sigma-Aldrich) and split across three 2-ml tubes. Each tube received 100 μl CD31-coupled Dynabeads (rat anti-mouse CD31, 10 μl/sample, 553370; BD Biosciences, RRID:AB_394816, conjugated to sheep anti-rat IgG Dynabeads, 50 μl/sample, 11035; Invitrogen) and was rotated for 30 min at room temperature. Tubes were placed on a DynaMag-2 magnet (12321D; Invitrogen), the supernatant was removed, and beads were washed twice by resuspension in secondary wash medium for magnetic capture. The three subsamples were pooled into one 2-ml tube, washed twice with ice-cold PBS, placed on the DynaMag-2 magnet, and the supernatant was used for mRNA extraction and cDNA conversion using a TaqMan Fast Advanced Cells-to-CT Kit (A35377; Invitrogen). cDNA was stored at −20°C until use. RT-qPCR was performed using TaqMan probes for *Slc2a1* (Mm00441480_m1) and *Gapdh* (Mm99999915_g1). For each technical replicate, 1.5 μl of cDNA was combined with 0.5-μl probe, 5 μl TaqMan Fast Advanced Master Mix, and 3 μl nuclease-free water. Three technical replicates were performed per sample per probe, and negative control and no-template controls were included for each probe. cDNA was amplified for 40 cycles, and fold-change in *Slc2a1* was calculated relative to the housekeeping gene *Gapdh* using the 2^(−ΔΔCt)^ method.

### Data and statistical analyses

For superfusion experiments, vessel diameters were determined from z-stacks of 3D volume recordings using the full-width at half-maximum (FWHM) method, where we averaged three measurements per vessel, either drawn across the cross-section of the PA or perpendicular to the capillary lumen. RBC flux was hand-counted and averaged from 1-s bins of 10-s line scan recordings using FIJI (RRID:SCR_002285) (Hariharan et al., 2022). PA diameter analyses for pressure-ejection and neurovascular coupling experiments were performed using a custom script on MATLAB (RRID:SCR_001622) that interpolates the FWHM of a line profile drawn across the arteriole (Isaacs et al., 2024). For quantification of immunohistochemistry images, peak intensities of GLUT1 and isolectin were measured from line profiles drawn across individual capillaries in Z projections of acquired images. Four capillaries per image were analyzed, and three to four images per slice were averaged as one technical replicate. For quantification of vessel density and pericyte numbers, *in vivo* Z stacks from a subset of experiments were processed in FIJI. Z stacks were smoothed, and vessel tree structure was analyzed using the Simple Neurite Tracer module within the Neuroanatomy plugin. First to eighth order capillary branches were traced based on the FITC-dextran channel, and TO-PRO-3–positive pericyte somas were hand-counted. Data were analyzed in GraphPad Prism (RRID:SCR_002798) using nested *t* tests or nested one-way ANOVA with post-hoc tests, as indicated in the respective figure legends. All data are presented as means ± SEM of biological replicates, and a P value ≤0.05 was considered significant. Sample sizes were estimated based on our previous experience with similar experiments. Experimenters were not blinded to experimental conditions.

### Online supplemental material

**Figure S1.**
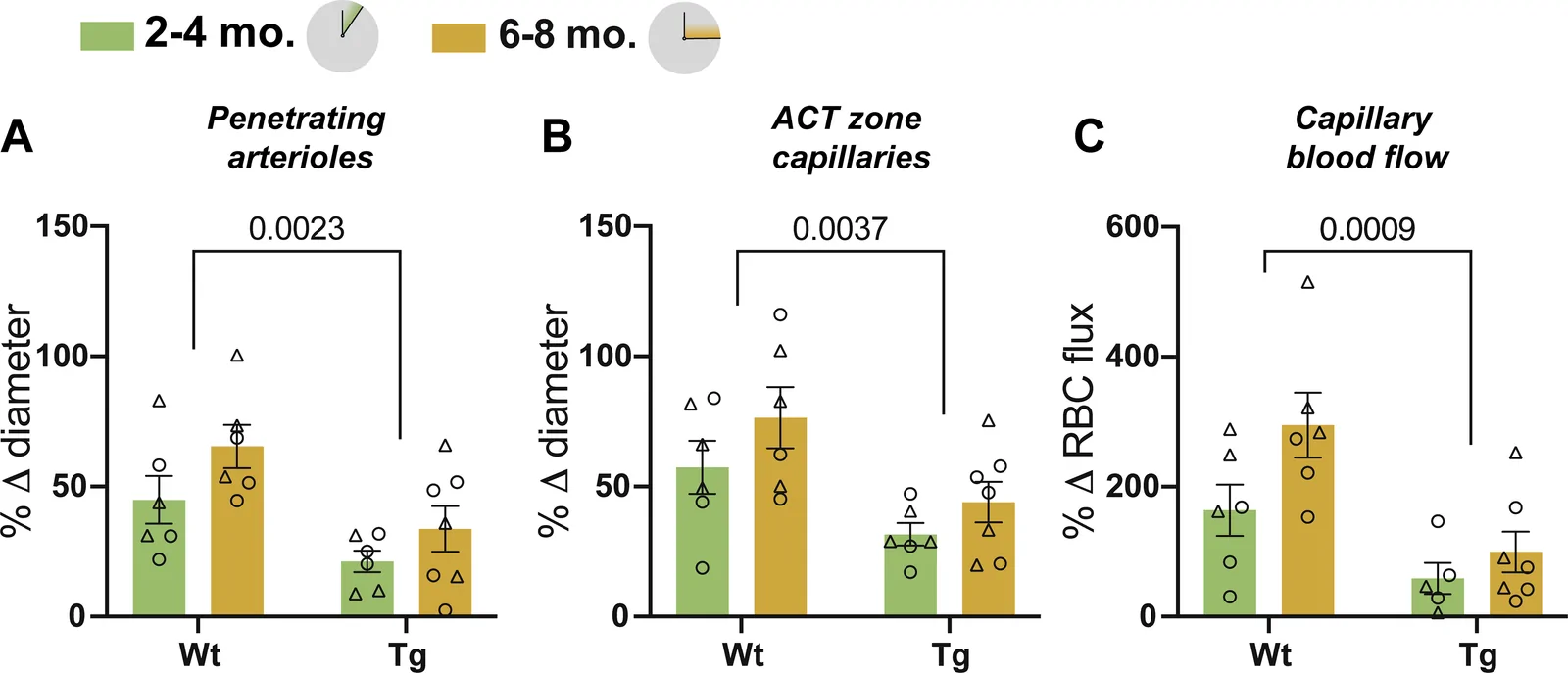
**Analyses of age and genotype effects on pinacidil-mediated blood flow control in 2–4- and 6–8-mo-old mice. (A–C)** Effects of age, genotype and their interaction on changes in (A) PA diameter (genotype: F_*1, 21*_ = 12.01, P = 0.0023; age: F_*1, 21*_ = 4.3, P = 0.0512; interaction: F_*1, 21*_ = 0.26, P = 0.6176), (B) ACT zone capillary diameter (genotype: F_*1, 21*_ = 10.65, P = 0.0037; age: F_*1, 21*_ = 3.12, P = 0.0918; interaction: F_*1, 21*_ = 0.14, P = 0.7077) and (C) RBC flux (genotype: F_*1, 20*_ = 15.28, P = 0.0009; age: F_*1, 20*_ = 5.02, P = 0.0366; interaction: F_*1, 20*_ = 1.37, P = 0.2559) to pinacidil superfusion. We detected significant effects of genotype across all three parameters, a significant effect of age on flux, where 6–8-mo-old mice had larger blood flow changes to pinacidil compared with 2–4-mo-olds, and no interaction between age and genotype for any of the parameters. Data were analyzed using two-way ANOVA (*n* = 6–7 mice per group) and are presented as biological means ± SEM. Male and female mice within each group are indicated by ο and Δ, respectively.

**Figure S2.**
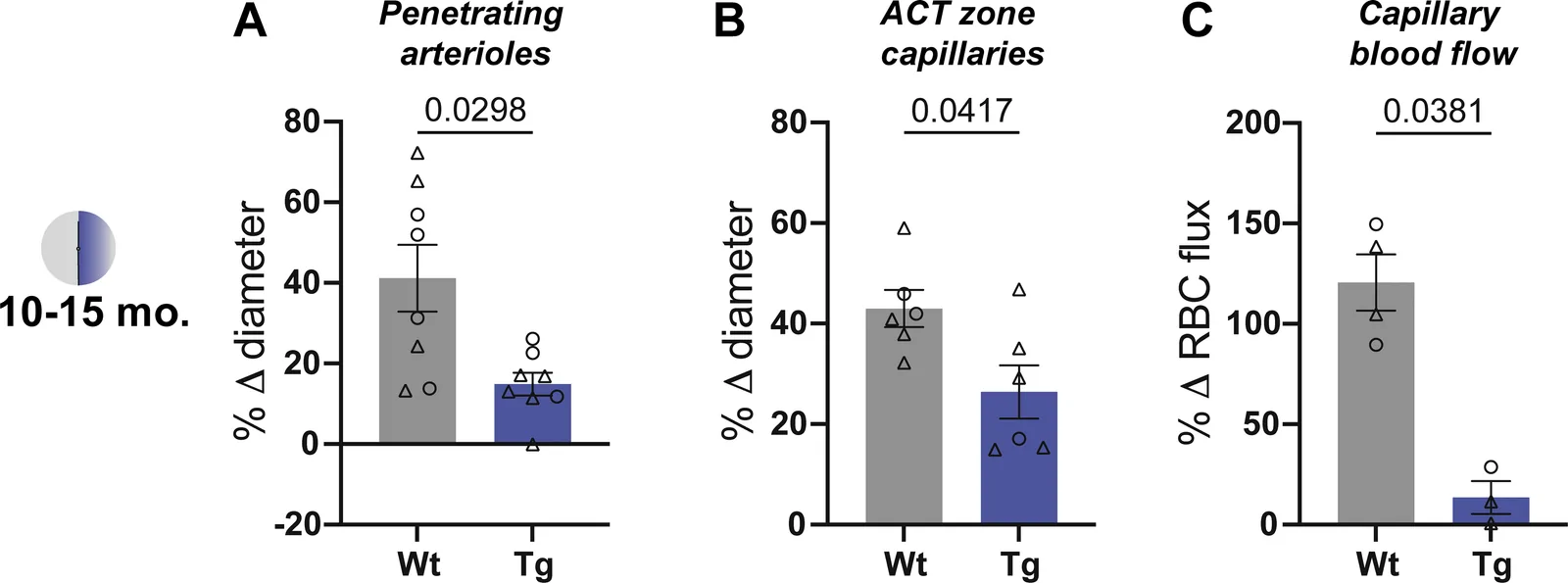
**Effects of pinacidil superfusion in 10–15-mo-old 5xFAD mice. (A–C)** Summary data showing significant impairments in pinacidil-mediated dilation of (A) PAs (*n* = 23 PAs from 8 Wt mice and 13 PAs from 8 Tg mice; *t*_*14*_ = 2.42, *F*_*1,14*_ = 5.85), (B) ACT zone capillaries (*n* = 70 capillaries from 6 Wt mice and 59 capillaries from 6 Tg mice; *t*_*10*_ = 2.34, *F*_*1,10*_ = 5.45), and (C) capillary RBC flux (*n* = 23 capillaries from 4 Wt mice and 11 capillaries from 3 Tg mice; *t*_*5*_ = 2.8, *F*_*1,5*_ = 7.83) in 10–15-mo-old Tg compared with Wt 5xFAD mice. Data were analyzed using nested *t* tests and are presented as biological means ± SEM. Male and female mice within each group are indicated by ο and Δ, respectively.

**Figure S3.**
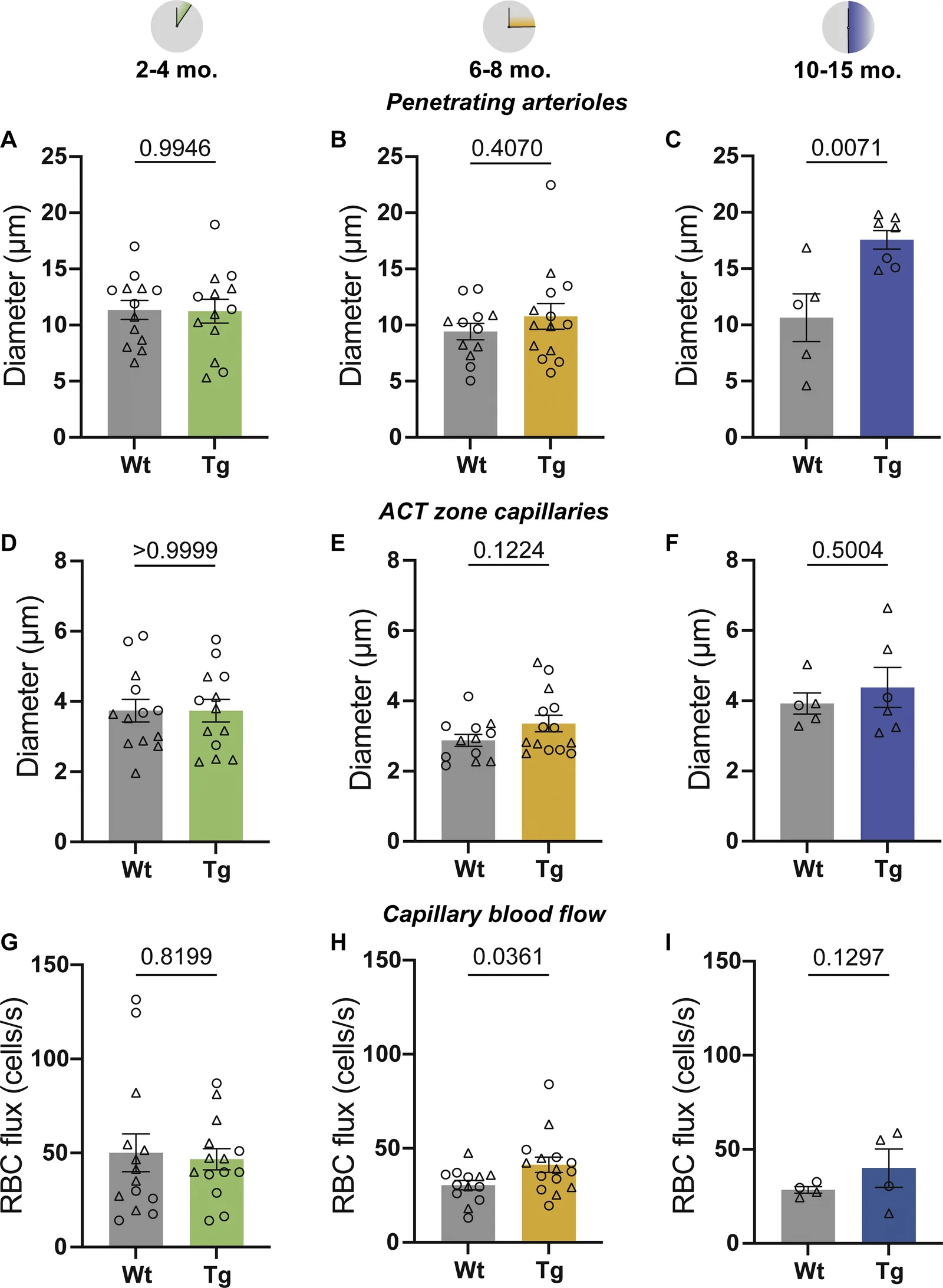
**Basal vessel diameters and RBC flux in 5xFAD mice. (A–C)** Summary data showing baseline PA diameters are not different between Wt and Tg 5xFAD mice at (A) 2–4 mo (*n* = 28 PAs from 13 Wt mice and 28 PAs from 13 Tg mice; *t*_*24*_ = 0.01, *F*_*1,24*_ = 4.65e-5) or (B) 6–8 mo (*n* = 29 PAs from 12 Wt mice and 33 PAs from 14 Tg mice; *t*_*24*_ = 0.84, *F*_*1,24*_ = 0.71) but (C) 10–15-mo-old Tg 5xFAD had significantly larger PA diameters compared with age-matched Wt controls (*n* = 11 PAs from 5 Wt mice and 12 PAs from 7 Tg mice; *t*_*10*_ = 3.37, *F*_*1,10*_ = 11.35). **(D–F)** Summary data showing baseline ACT zone capillary diameters are not different between Wt and Tg 5xFAD mice at (D) 2–4 mo (*n* = 171 capillaries from 13 Wt mice and 172 capillaries from 13 Tg mice; *t*_*24*_ = 9.8 × 10^−5^, *F*_*1,24*_ = 9.61 × 10^−9^), (E) 6–8 mo (*n* = 161 capillaries from 12 Wt mice and 176 capillaries, from 14 Tg mice; *t*_*24*_ = 1.6, *F*_*1,24*_ = 2.56), or (F) 10–15 mo (*n* = 57 capillaries from 5 Wt mice and 59 capillaries from 6 Tg mice; *t*_*9*_ = 0.7, *F*_*1,9*_ = 0.49). **(G–I)** Summary of baseline flux showing no significant difference between Wt and Tg 5xFAD mice at (G) 2–4 mo (*n* = 60 capillaries from 14 Wt mice and 68 capillaries from 14 Tg mice; *t*_*26*_ = 0.23, *F*_*1,26*_ = 0.05) or (I) 10–15 mo (*n* = 24 capillaries from 4 Wt mice and 14 capillaries from 4 Tg mice; *t*_*6*_ = 1.76, *F*_*1,6*_ = 3.08) but (H) 6–8-mo-old Tg mice had significantly elevated RBC flux compared with Wt littermates (*n* = 61 capillaries from 13 Wt mice and 57 capillaries from 15 Tg mice; *t*_*26*_ = 2.21, *F*_*1,26*_ = 4.89). Data were analyzed using nested *t* tests and are presented as biological means ± SEM. Male and female mice within each group are indicated by ο and Δ, respectively.

**Figure S4.**
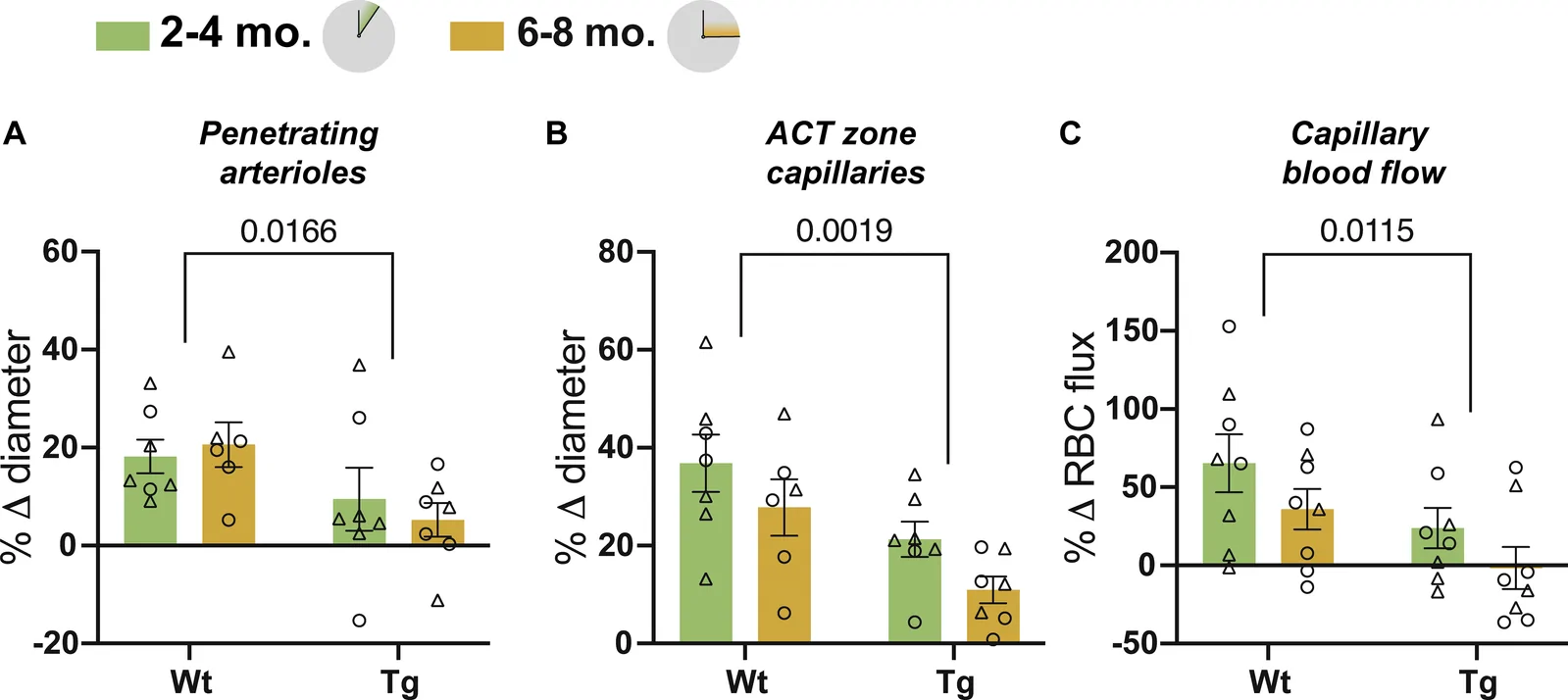
**Effects of age and genotype on BAY-876 superfusion-mediated changes in vessel diameters and blood flow. (A–C)** Effects of age, genotype and their interaction on changes in (A) PA diameter (genotype: F_*1, 23*_ = 6.68, P = 0.0166; age: F_*1, 23*_ = 0.04, P = 0.8457; interaction: F_*1, 23*_ = 0.51, P = 0.4819), (B) ACT zone capillary diameter (genotype: F_*1, 23*_ = 12.27, P = 0.0019; age: F_*1, 23*_ = 4.44, P = 0.0461; interaction: F_*1, 23*_ = 0.02, P = 0.8853), and (C) RBC flux (genotype: F_*1, 23*_ = 7.31, P = 0.0115; age: F_*1, 23*_ = 3.52, P = 0.0713; interaction: F_*1, 23*_ = 0.02, P = 0.895) to BAY-876 superfusion show a significant effect of genotype across all three parameters, a significant effect of age on ACT zone diameter changes and no interaction between age and genotype for any of the parameters. Biological means were analyzed using two-way ANOVA (*n* = 6–8 mice per group), and data are presented as biological means ± SEM. Male and female mice within each group are indicated by ο and Δ, respectively.

**Figure S5.**
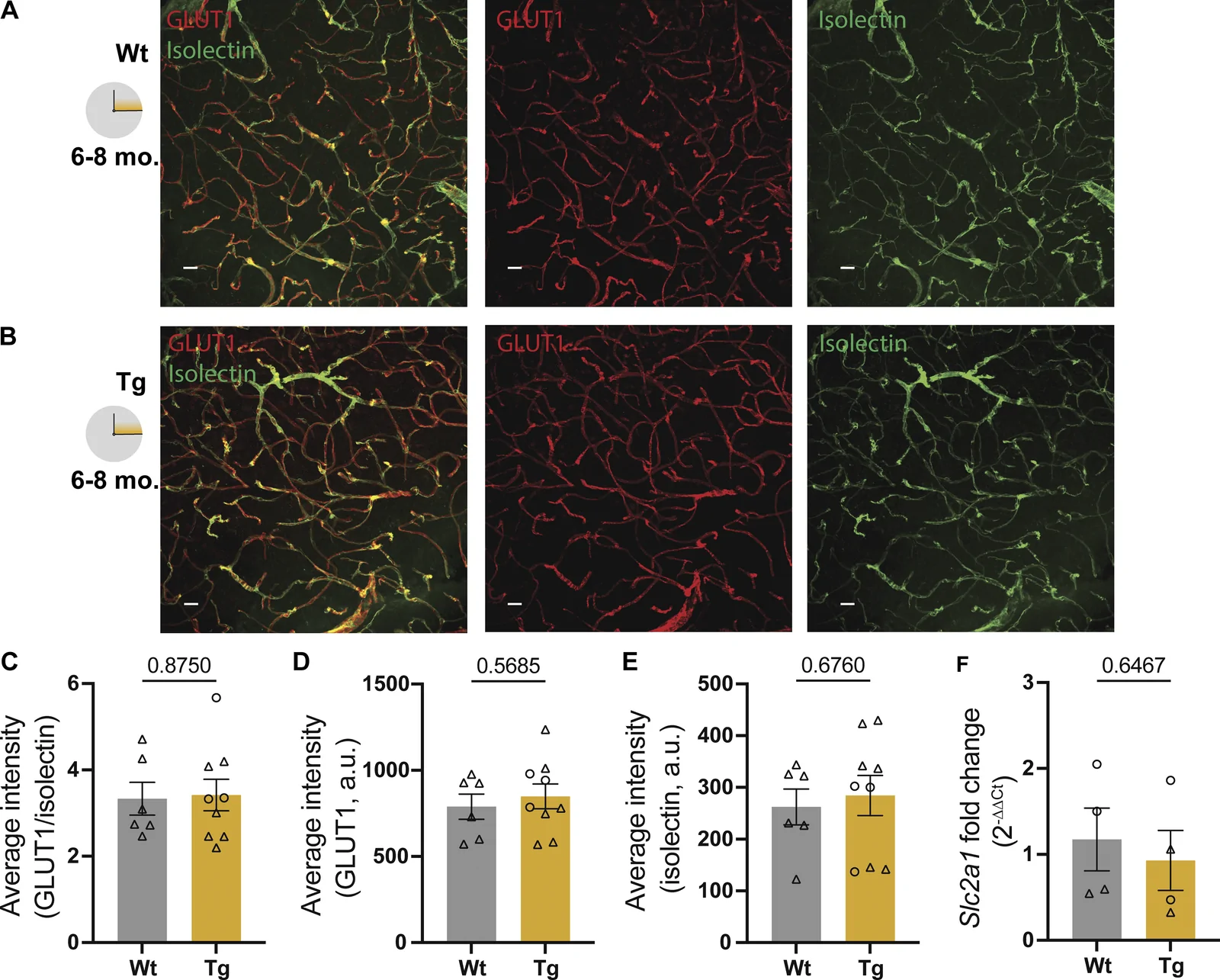
**GLUT1 expression and mRNA analyses from isolated microvessels in 5xFAD mice. (A and B)** Example Z-projection images from 6–8-mo-old (A) Wt and (B) Tg 5xFAD mice showing GLUT1 and isolectin immunofluorescence labelling. **(C–E)** Summary data showing that average intensities of (C) GLUT1 normalized to isolectin (*t*_*12*_ = 0.16, *F*_*8,5*_ = 1.4), (D) GLUT1 alone (*t*_*12.2*_ = 0.59, *F*_*8,5*_ = 1.45), and (E) isolectin alone (*t*_*12.8*_ = 0.43, *F*_*8,5*_ = 1.89) were not significantly different between Wt and Tg 5xFAD mice at 6–8 mo of age (*n* = 6 slices, from 2 Wt mice and 9 slices, from 3 Tg mice; a.u., arbitrary units). **(F)** GLUT1 (*Slc2a1*) mRNA relative to the housekeeping gene GAPDH (*Gapdh*) was not significantly different between 6 and 8-mo-old Wt and Tg 5xFAD mice (*t*_*0.48*_ = 5.99, *F*_*3,3*_ = 1.1). Data were analyzed using Welch’s *t* tests and are presented as technical (C–E) or biological replicates (F) ± SEM. Male and female mice within each group are indicated by ο and Δ, respectively.

**Figure S6.**
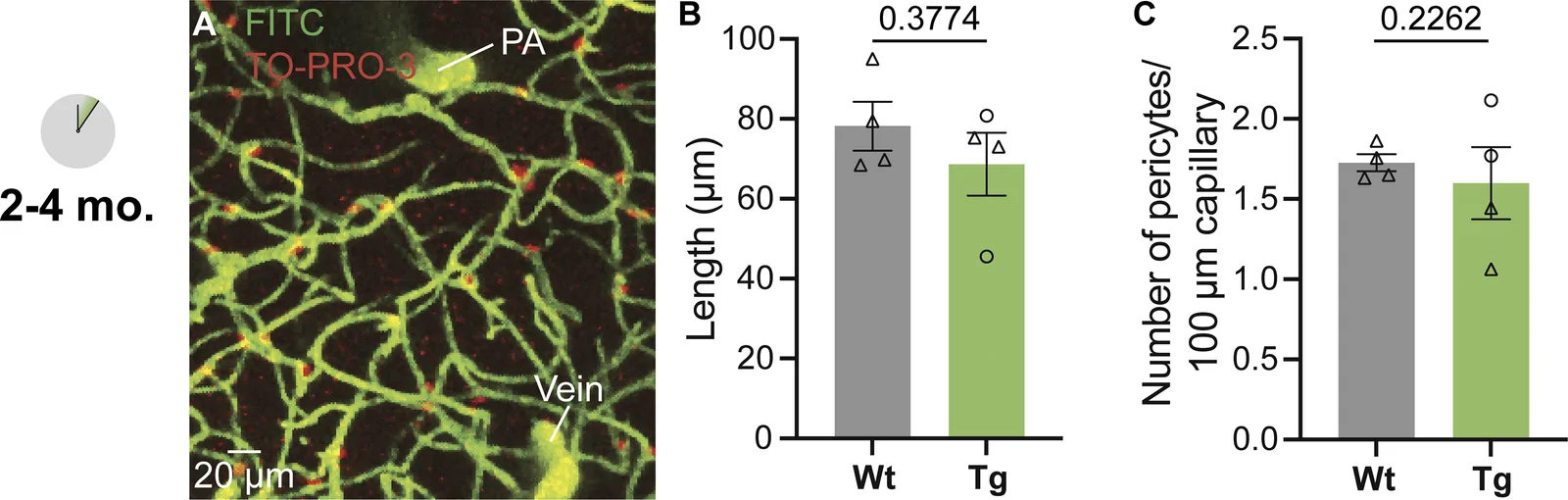
**Morphological analyses of capillaries in 2–4-mo-old 5xFAD mice.** **(A)** A typical *in vivo* Z-stack from a 2–4-mo-old Tg 5xFAD mouse used for morphological analysis, showing FITC-containing vasculature and TO-PRO-3–stained pericytes on capillaries. **(B and C)** Summary data demonstrating (B) vessel length (*t*_*6*_ = 0.95, *F*_*8,5*_ = 0.91) and (C) TO-PRO-3-positive pericyte numbers (*t*_*6*_ = 1.35, *F*_*8,5*_ = 0.23) measured from first to eighth order capillaries were not significantly different between 2–4-mo-old Wt and Tg 5xFAD mice (*n* = 4 mice/group). Data were analyzed using nested *t* tests and are presented as biological means ± SEM. Male and female mice within each group are indicated by ο and Δ, respectively.

Fig. S1 shows age and genotype effects on pinacidil-mediated blood flow control in 2–4- and 6–8-mo-old mice. Fig. S2 shows the effects of pinacidil superfusion in 10–15-mo-old 5xFAD mice. Fig. S3 shows basal vessel diameters and RBC flux in 5xFAD mice. Fig. S4 shows the effects of age and genotype on BAY-876 superfusion-mediated changes in vessel diameters and blood flow. Fig. S5 shows GLUT1 expression and mRNA analyses from isolated microvessels in 5xFAD mice. Fig. S6 shows morphological vessel analyses from 2–4-mo-old Wt and Tg 5xFAD mice.

## Results

### Impairments in K_ATP_-mediated dilation and hyperemia in 5xFAD mice start early and are long-lasting

**Figure 1.**
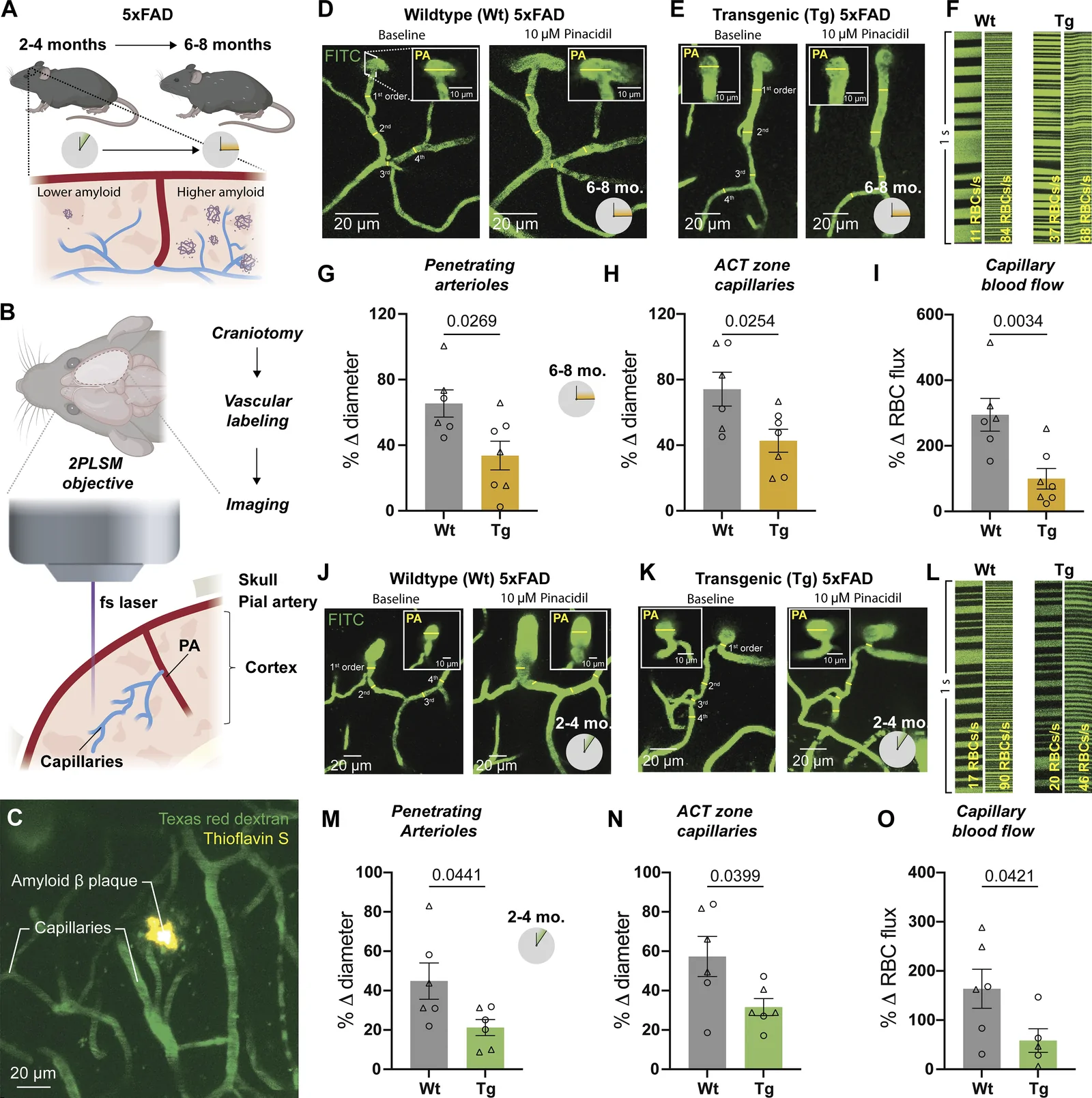
**AD mutations impair K**_**ATP**_ **channel–mediated dilation and hyperemia *in vivo* in 5xFAD mice. (A)** Illustration showing the two primary age points used in this study that reflect early (2–4 mo) and moderate (6–8 mo) stages of disease progression in 5xFAD mice. **(B)** Experimental setup of *in vivo* imaging of cerebral hemodynamics. **(C)** Z-projection of a representative *in vivo* vascular network showing an amyloid β plaque stained with thioflavin S. **(D and E)** Z-projections of representative *in vivo* 3D volume acquisitions showing diameters of PA and ACT zone capillaries at (left) baseline and (right) after the application of 10 μM pinacidil. Pinacidil-mediated dilation is intact in (D) Wt but impaired in (E) Tg 5xFAD mice at 6–8 mo of age. Inset shows the PA in a single plane, and yellow lines indicate diameters of different vessels at baseline for comparison across images. **(F)** Line-scan kymographs of RBC flux measured from equal to or higher-than fifth-order capillaries at baseline (left) and after (right) 10 μM pinacidil superfusion showing a larger increase in capillary blood flow in 6–8-mo-old Wt than in Tg 5xFAD mice of the same age. **(G–I)** Summary data showing significant impairments in pinacidil-mediated dilation of (G) PAs (*n* = 17 Wt PAs, 6 mice and 20 Tg PAs, 7 mice; *t*_*11*_ = 2.55, *F*_*1,11*_ = 6.51), (H) ACT zone capillaries (*n* = 80 capillaries from 6 Wt mice and 92 capillaries from 7 Tg mice; *t*_*11*_ = 2.37, *F*_*1,11*_ = 5.61), and (I) capillary RBC flux (*n* = 31 capillaries from 6 Wt mice and 28 capillaries from 7 Tg mice; *t*_*11*_ = 3.71, *F*_*1,11*_ = 13.76) in 6–8–mo-old Tg 5xFAD mice compared with Wt littermates. **(J and K)** Representative Z-projection showing diameters of PA and ACT zone capillaries at baseline (left) and after the application of 10 μM pinacidil (right). Pinacidil-mediated dilation is intact in (J) Wt but impaired in (K) Tg 5xFAD mice at 2–4 mo of age. Yellow lines indicate diameters of different vessels at baseline for comparison, and single planes of PAs are shown in the inset. **(L)** RBC flux kymographs at baseline (left) and after 10 μM pinacidil superfusion (right) showing a large increase in capillary blood flow in 2–4-mo-old Wt mice, which is blunted in Tg 5xFAD mice. **(M–O)** Summary data showing significant impairments in pinacidil-mediated dilation of (M) PAs (*n* = 14 PAs from 6 Wt mice and 13 PAs from 6 Tg mice; *t*_*10*_ = 2.3, *F*_*1,10*_ = 5.3), (N) ACT zone capillaries (*n* = 76 capillaries from 6 Wt mice and 78 capillaries from 6 Tg mice; *t*_*10*_ = 2.36, *F*_*1,10*_ = 5.58), and (O) capillary RBC flux (*n* = 27 capillaries from 6 Wt mice and 22 capillaries from 6 Tg mice; *t*_*10*_ = 2.33, *F*_*1,10*_ = 5.42) in 2–4-mo-old Tg 5xFAD mice compared with Wt littermates. Data were analyzed using nested *t* tests and are presented as biological means ± SEM. Male and female mice within each group are indicated by ο and Δ, respectively.

The subunits of the vascular isoform of the K_ATP_ channel—the inward-rectifier K_ir_6.1 (encoded by *Kcnj8*) and sulfonylurea receptor 2 (SUR2; encoded by *Abcc9*)—are among the most highly expressed of all ion channel components by brain thin-strand pericytes (Hariharan et al., 2020; He et al., 2018; Vanlandewijck et al., 2018). *In vivo*, activation of these K_ATP_ channels with pinacidil can produce robust increases in capillary blood flow and dilate upstream PAs and ACT zone capillaries (Hariharan et al., 2022). To initially assess the effect of familial AD mutations on K_ATP_ function, we applied 10 μM pinacidil to the brain surface of 6–8-mo-old 5xFAD and Wt mice. Here, we found significantly diminished dilation of the PA and first- to fourth-order capillaries of the ACT zone to this maneuver, and RBC flux increases measured in fifth order or higher capillaries in 5xFAD mice were blunted compared with Wt controls (Fig. 1, A–I). To explore the onset of this K_ATP_ dysfunction, we repeated these measurements in younger (2–4-mo-old) mice. Surprisingly, even at this early time point, we found equally marked reductions in pinacidil-induced dilation of PAs and ACT zone capillaries and blood flow elevations in mice harboring *APP* and *PSEN1* mutations (Fig. 1, J–O). On average, pinacidil-induced dilation of PAs and capillaries in Tg 5xFAD mice decreased by ∼50% at 2–4 mo and ∼45% at 6–8 mo compared with Wt controls, whereas the deficits in RBC flux elevations were slightly more severe—down ∼66% at both age points. When analyzing the effects of genotype and age on pinacidil-mediated changes in PA and capillary diameters and RBC flux, we found no interaction between the two, but we observed a significant effect of genotype on all three parameters and an effect of age on capillary flux (Fig. S1, A–C).

To evaluate if these impairments to pinacidil superfusion persisted or became more severe beyond 6–8 mo in 5xFAD mice, we also analyzed responses of PA and ACT zone diameters and capillary blood flow to pinacidil superfusion at 10–15 mo, where we found similar deficits to those observed at earlier ages (Fig. S2, A–C). Collectively, these data demonstrate an early and long-lasting impairment of blood flow control by K_ATP_ channels in 5xFAD mice.

To assess whether familial AD mutations affected baseline vascular dynamics, we compared vessel diameters and capillary blood flow at rest across age groups. Baseline diameters of PAs and ACT zone capillaries were not significantly different between 5xFADs and Wt littermates at 2–4 or 6–8 mo (Fig. S3, A, B, D, and E). At the older age of 10–15 mo, 5xFAD mice had significantly larger PAs, while ACT zone capillaries were similar between groups (Fig. S3, C and F). This suggests that a deficit in PA myogenic tone, which provides the autoregulatory ability of blood vessels to constrict in response to increased intravascular pressure, emerges in 5xFAD mice as they progressively age but does not manifest at earlier time points. Baseline blood flow was similar between groups at 2–4 mo, although we noted a significant elevation in 5xFADs at 6–8 mo (Fig. S3, G and H), contrary to other reports of reduced blood flow in 5xFAD mice at this age (Cruz Hernández et al., 2019). Although not statistically significant, a similar trend was observed in 10–15-mo-old mice (Fig. S3 I). While we did not measure capillary stalling behavior in these experiments, our finding of increased basal flux in 5xFADs could reflect sampling from “flowing” capillaries, which may carry more RBCs to compensate for stalled capillaries elsewhere in the same network (Cruz Hernández et al., 2019).

### Effects of familial AD mutations on the pericyte energy switch mechanism

**Figure 2.**
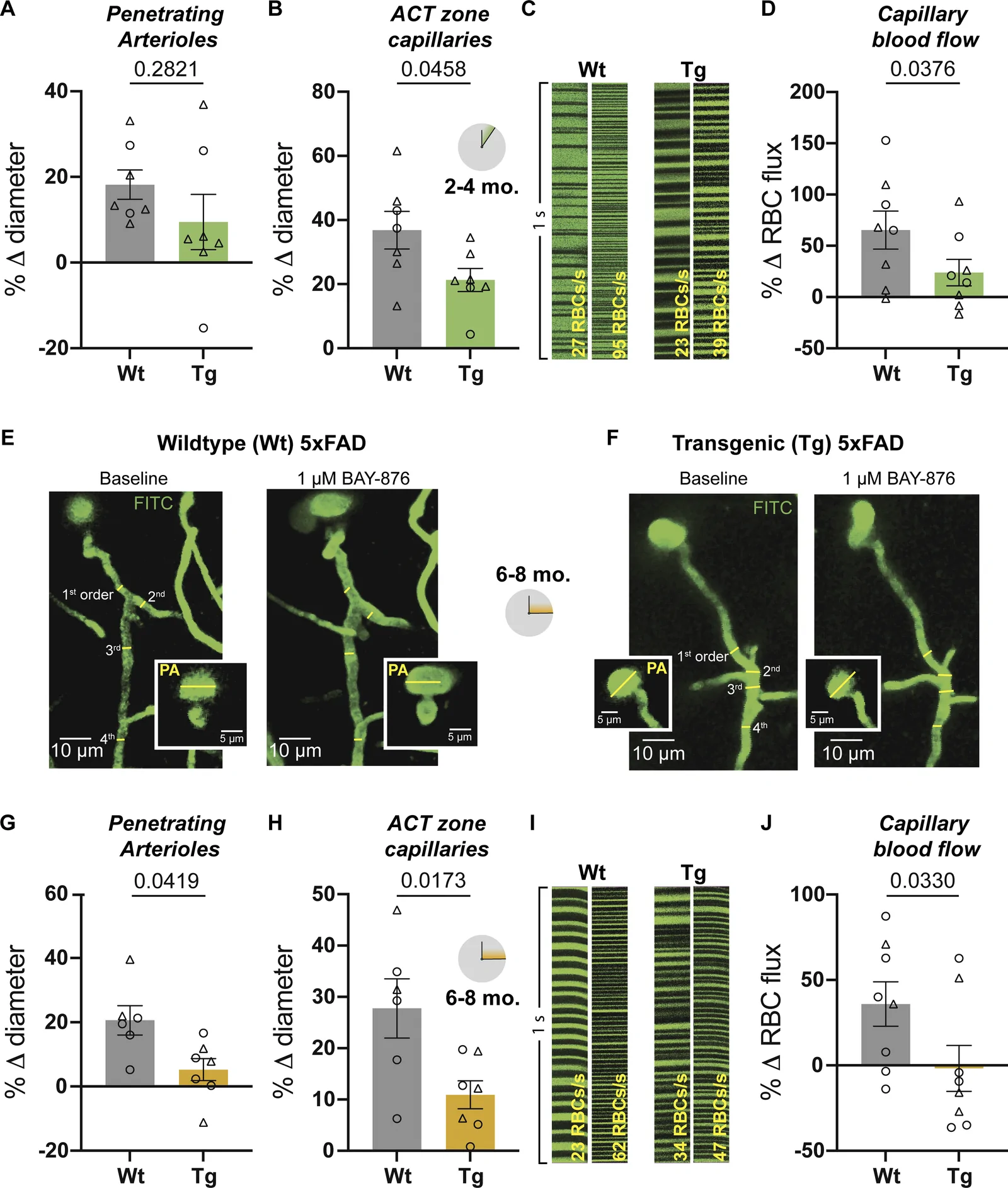
**Pericyte K**_**ATP**_ **channel metabolic sensing is disrupted in 5xFAD mice. (A and B)** Summary data showing (A) PA dilation is intact (*n* = 14 PAs from 7 Wt mice and 15 PAs from 7 Tg mice; *t*_*12*_ = 1.13, *F*_*1,12*_ = 1.27) but (B) ACT zone capillary dilation (*n* = 96 capillaries from 7 Wt mice and 90 capillaries from 7 Tg mice; *t*_*12*_ = 2.23, *F*_*1,12*_ = 4.96) is significantly impaired after 1 μM BAY-876 application in 2–4-mo-old Tg 5xFAD mice compared with Wt littermate controls. **(C)** Representative RBC flux kymographs at baseline (left) and after BAY-876 superfusion (right) indicating a larger increase in blood flow in 2–4-mo-old Wt mice compared with Tg 5xFAD mice. **(D)** Summary data showing significantly lower RBC flux change in response to BAY-876 in 2–4-mo-old Tg 5xFAD mice compared with Wt littermates (*n* = 36 capillaries from 8 Wt mice and 43 capillaries from 8 Tg mice; *t*_*14*_ = 2.3, *F*_*1,14*_ = 5.28). **(E and F)** Representative images showing that GLUT1 blockade with 1 μM BAY-876 dilates PAs and ACT zone capillaries in 6–8-mo-old (E) Wt but not in (F) Tg 5xFAD mice. Yellow lines indicate vessel diameters at baseline for comparison, and insets show the PA diameter in a single plane. **(G and H)** Summary data demonstrating significantly impaired dilation of (G) PAs (*n* = 12 PAs from 6 Wt mice and 14 PAs from 7 Tg mice; *t*_*11*_ = 2.3, *F*_*1,11*_ = 5.3), and (H) ACT zone capillaries (*n* = 84 capillaries from 6 Wt mice and 87 capillaries from 7 Tg mice; *t*_*11*_ = 2.8, *F*_*1,11*_ = 7.84) in 6–8-mo-old Tg 5xFAD mice compared with Wt littermates in response to 1 μM BAY-876 superfusion. **(I)** Kymographs showing that the BAY-876–mediated increase in RBC flux is more robust in Wt mice (left) compared with Tg 5xFAD mice (right) at 6–8 mo of age. **(J)** Summary data showing significantly decreased RBC flux change to BAY-876 in 6–8-mo-old Tg 5xFAD mice compared with Wt littermates (*n* = 29 capillaries from 8 Wt mice and 27 capillaries from 8 Tg mice; *t*_*14*_ = 2.37, *F*_*1,14*_ = 5.59). Data were analyzed using nested *t* tests and are presented as biological means ± SEM. Male and female mice within each group are indicated by ο and Δ, respectively.

As their name suggests, K_ATP_ channels couple intracellular metabolic changes to membrane electrical activity (Akrouh et al., 2009), and we have shown a role for these channels in the capillary bed of the brain where they enable thin-strand pericytes to sense changes in local glucose availability and respond to deficits by generating hyperpolarizing electrical signals that produce robust increases in blood flow (Hariharan et al., 2022). This mechanism may be highly relevant for tissue homeostasis in AD, where glucose handling and dynamics are known to be profoundly dysregulated (Kyrtata et al., 2021; Mosconi, 2005; Mosconi, 2013; Simpson et al., 1994). GLUT-1 is expressed at the blood–brain barrier (He et al., 2018; Vanlandewijck et al., 2018), and we have shown that blocking glucose import via this protein through surface application of 1 μM BAY-876 flips the thin-strand pericyte K_ATP_ channel-dependent energy switch to increase local blood flow (Hariharan et al., 2022). Based on our initial finding that K_ATP_-mediated control of blood flow was already impaired in 2–4-mo-old 5xFAD mice, we used this approach to probe whether the pericyte energy switch is also affected at this time point. BAY-876-mediated dilation of PAs was intact (albeit with a strong trend toward a decrease of ∼50% in 5xFADs), while the dilation of ACT zone capillaries and rise in RBC flux were disrupted at 2–4 mo (Fig. 2, A–D). At 6–8 mo, all three of these parameters were significantly diminished (Fig. 2, E–J) in 5xFAD mice compared with Wt age-matched controls. Two-way ANOVA revealed significant effects of genotype across all measured parameters, and a significant effect of age on ACT zone capillary diameter changes such that 2–4-mo-old mice had greater responses to BAY-876 than 6–8-mo-old mice (Fig. S4, A–C). No interactions between genotype and sex were observed for any of the three variables.

To test whether changes in GLUT1 levels could underlie these diminished BAY-876 effects, we measured GLUT1 expression at the blood–brain barrier in 6–8-mo-old mice (when Aβ pathology is more severe) using immunohistochemistry. At this age, the average intensities of GLUT1 and the basement membrane marker isolectin were similar across Tg 5xFADs and Wt littermates (Fig. S5, A–E), indicating that the observed impairments are attributable to dysfunctional electro-metabolic signaling as opposed to loss of substrate transport capability. This correlated with RT-qPCR analysis of mRNA levels of GLUT1 (*Slc2a1*) in isolated vessels from barrel cortices of 6–8-mo-old mice, which revealed no differences between 5xFAD mice and their Wt littermates (Fig. S5 F). Together, these data suggested that transcriptional and protein level changes of GLUT1 in 5xFAD mice were not the underlying cause of vascular impairments we observed. This led us to examine the function of K_ATP_ channel control of thin-strand pericyte membrane potential in 5xFAD mice in detail.

### Control of pericyte membrane potential by K_ATP_ channels is intact at 2–4 mo in 5xFAD mice but is defunct by 6–8 mo of age

**Figure 3.**
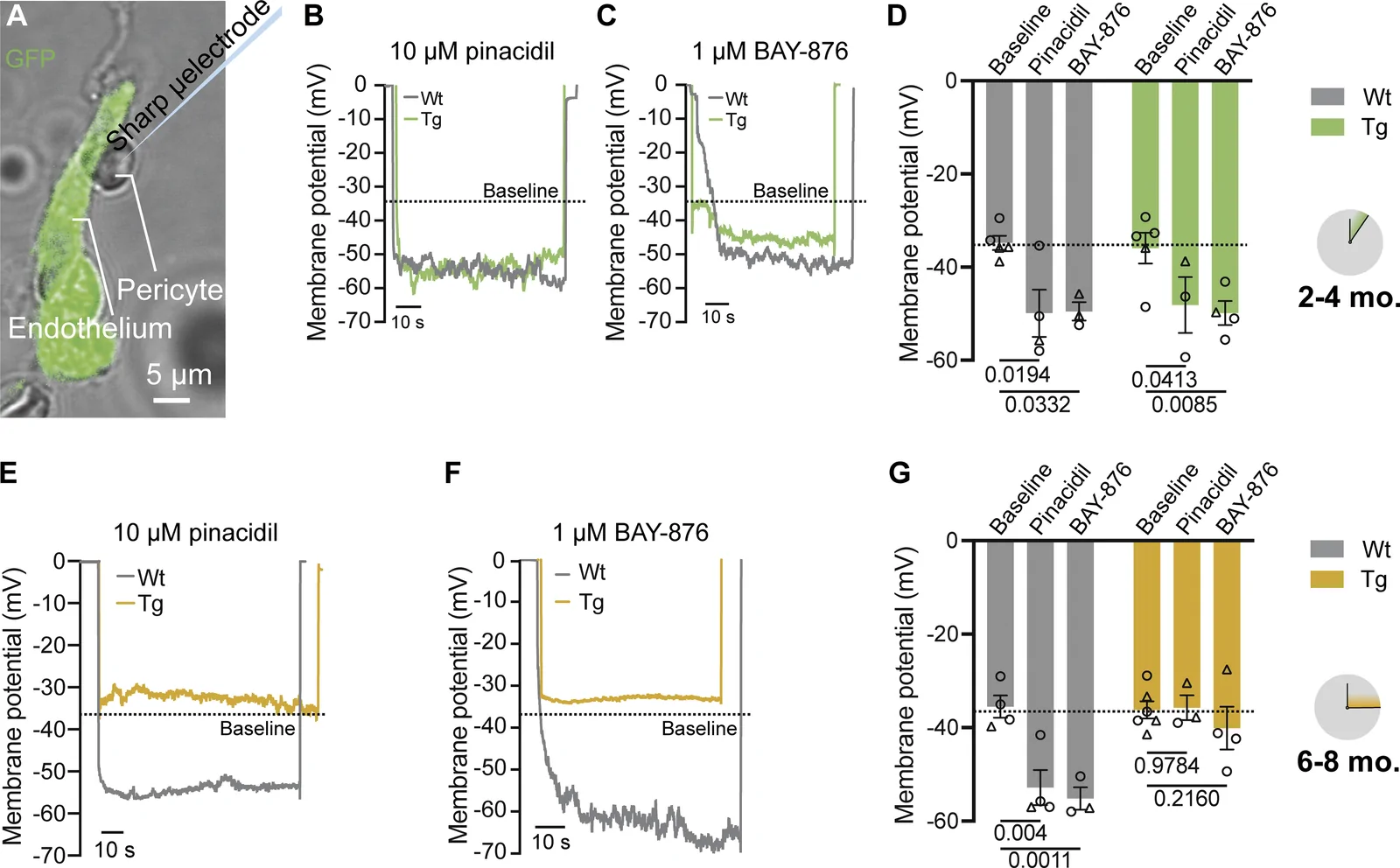
**Pericyte K**_**ATP**_ **channel–mediated changes in membrane potential are intact in young 5xFAD mice and lost by 6–8 mo of age. (A)** Strategy to identify pericytes in 5xFAD mice. Image showing AAV(BR1)-CAG-GFP-labelled endothelium and a nonfluorescent bump-on-a-log pericyte cell body identified by phase-contrast microscopy. **(B and C)** Representative traces from sharp microelectrode impalements showing similar membrane potential responses of 2–4-mo-old Wt and Tg 5xFAD mice in response to 10 μM pinacidil (B) and 1 μM BAY-876 (C). Dotted lines indicate the mean baseline membrane potential of 2–4-mo-old Wt mice. **(D)** Summary data illustrating significant hyperpolarization of pericyte membrane potential in 2–4-mo-old Wt and Tg 5xFAD mice in response to pinacidil and BAY-876 (*n* = 9–17 cells, 3–5 Wt mice; baseline vs. pinacidil: *t*_*18*_ = 3.2, baseline vs. BAY-876: *t*_*18*_ = 2.96; *n* = 14–20 cells, 3–5 Tg mice; baseline vs. pinacidil: *t*_*18*_ = 2.86, baseline vs. BAY-876: *t*_*18*_ = 3.58; *F*_*5,18*_ = 5.63). Mean baseline membrane potential of 2–4-mo-old Wt mice is indicated by the dotted line. **(E and F)** Representative traces showing that membrane potential responses of 6–8-mo-old Tg 5xFAD mice to bath application of 10 μM pinacidil (E) and 1 μM BAY-876 (F) are impaired compared with age-matched Wt littermates. Dotted lines indicate mean baseline membrane potential of 6–8-mo-old Wt mice. **(G)** Summary data showing that membrane potential responses to K_ATP_ activators pinacidil and BAY-876 are intact in Wt but defunct in Tg 5xFAD mice at the age of 6–8 mo (*n* = 9–14 cells, 3–4 Wt mice; baseline vs. pinacidil: *t*_*18*_ = 3.29, baseline vs. BAY-876: *t*_*18*_ = 3.89; *n* = 10–21 cells, 3–6 Tg mice; baseline vs. pinacidil: *t*_*18*_ = 0.03, baseline vs. BAY-876: *t*_*18*_ = 1.16; *F*_*5,18*_ = 7.14). Mean baseline membrane potential of 6–8-mo-old Wt mice is indicated by the dotted line. Data were analyzed using nested one-way ANOVA and Sidak’s multiple comparisons test and presented as biological means ± SEM. Male and female mice within each group are indicated by (ο) and (Δ), respectively.

To correlate the observed *in vivo* impairments in K_ATP_ and energy switch control of capillary blood flow and vessel dilation with pericyte electrical activity in 5xFAD mice, we next directly measured the magnitude of hyperpolarization generated by pinacidil or BAY-876 in thin-strand pericytes. Use of an AAV-based strategy to selectively transfect brain endothelial cells with eGFP enabled us to identify thin-strand pericytes as nonfluorescent cells sitting atop eGFP-labeled endothelium via combined phase-contrast and fluorescence microscopy (Fig. 3 A) (Xiang et al., 2026). The baseline membrane potential of impaled thin-strand pericytes from 2–4-mo-old Wt mice was −34.8 ± 1.5 mV (*n* = 17 cells from 5 mice), similar to previous observations (Hariharan et al., 2022; Sancho et al., 2022), and this was not significantly different from the baseline membrane potential of 2–4-mo-old Tg mice (−35.9 ± 3.3 mV; 14 cells from 5 mice) or from the baseline of 6–8-mo-old Wt (−35.5 ± 2.4; 9 cells from 4 mice) or Tg mice (−36.2 ± 1.8 mV; 12 cells from 6 mice). We showed previously that pericyte membrane potential is hyperpolarized in the presence of 10 μM pinacidil by an average of 23 mV in healthy 2–3-mo-old C57BL/6 mice, and by ∼15 mV in response to 1 μM BAY-876, due to the activation of K_ATP_ channels in both cases (Hariharan et al., 2022). At 2–4 mo, we found that 5xFAD and Wt mice had similar and significant hyperpolarizing responses to pinacidil (by an average of 15 mV; Fig. 3, B and D) and BAY-876 (by an average of 14 mV; Fig. 3, C and D), indicating that K_ATP_ channel activity was intact at this time point (Fig. 3 D). However, at 6–8 mo neither pinacidil (Fig. 3, E and G) nor BAY-876 (Fig. 3, F and G) were capable of eliciting hyperpolarization of thin-strand pericytes in Tg 5xFADs (Fig. 3 G).

Taken together, our data suggest that while the functional outcome of K_ATP_ activity is impaired *in vivo* at 2–4 mo, defects in K_ATP_ function in thin-strand pericytes alone are not sufficient to explain this issue. Rather, defects in membrane potential responses to the K_ATP_ opener pinacidil and the metabolic switch activator BAY-876 appear later and are detectable by 6–8 mo of age in 5xFAD mice. These observations thus prompted us to examine the electrical signal transmission capabilities of capillaries in young 5xFAD mice to determine whether deficits here undermine K_ATP_-mediated signaling.

### Loss of capillary K_ir_2.1-dependent electrical signaling precedes and exacerbates pericyte K_ATP_ channel dysfunction in 5xFAD mice

**Figure 4.**
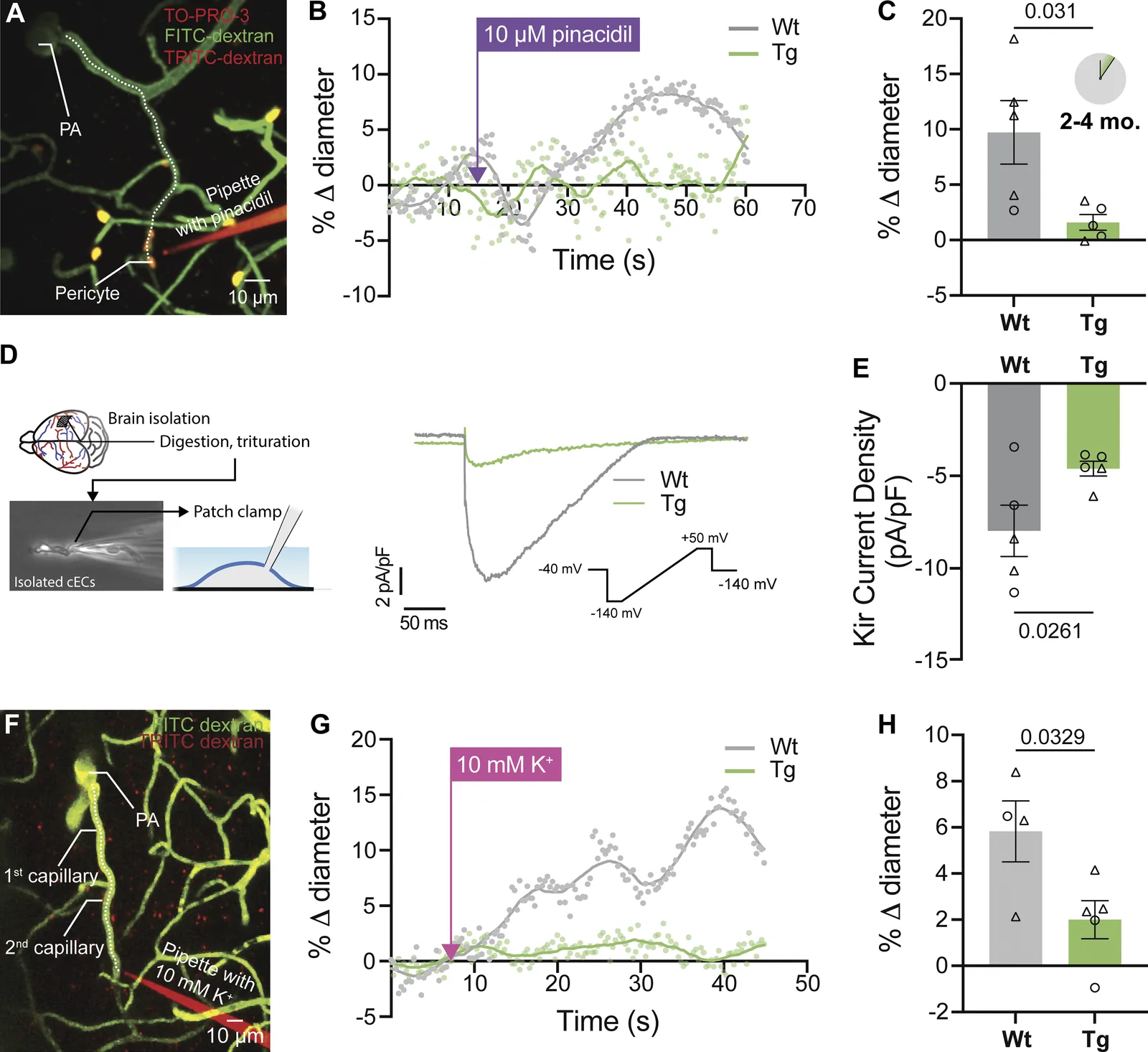
**Transmission of retrograde signals along the capillary–PA axis is lost in young 5xFAD mice. (A)** *In vivo* experimental setup showing a pipette containing pinacidil and TRITC-dextran positioned next to a fifth-order pericyte stained with TO-PRO-3. **(B)** Example traces of PA diameter changes in response to selective application of 10 μM pinacidil to a thin-strand pericyte. The connected upstream PA dilates in 2–4-mo-old Wt mice, and this response is reduced in Tg 5xFAD mice. **(C)** Summary data showing that PA dilation after pinacidil stimulation of a downstream pericyte *in vivo* is significantly smaller in 2–4-mo-old Tg 5xFAD mice compared with Wt littermate controls (*n* = 9 PAs from 5 Wt mice and 6 PAs from 5 Tg mice, *t*_*8*_ = 2.61, *F*_*1,8*_ = 6.83). **(D)** Left: Capillary isolation and identification of cECs for whole-cell patch-clamp electrophysiology. Right: Example K_ir_2.1 currents from freshly isolated cECs showing smaller currents in cells from 2–4-mo-old Tg 5xFAD mice compared with Wt littermates. **(E)** Summary data demonstrating significantly smaller cEC K_ir_2.1 currents in 2–4-mo-old Tg 5xFAD mice compared with Wt controls (*n* = 13 cells from 5 Wt mice and 14 cells from 5 Tg mice; *t*_*8*_ = 2.72, *F*_*1,8*_ = 7.42). **(F)** Z-projection showing a pipette containing 10 mM K^+^ and TRITC-dextran positioned next to a second-order capillary *in vivo*. **(G)** Example traces of PA dilation in response to application of 10 mM K^+^ onto a connected, downstream second- or third-order capillary of the ACT zone, showing a larger PA dilation in a 2–4-mo-old Wt mouse compared with an age-matched Tg 5xFAD mouse. **(H)** Summary of PA diameter changes demonstrating that 2–4-mo-old Tg 5xFAD mice have significantly blunted PA responses to 10 mM K^+^ capillary application *in vivo* compared with Wt littermates (*n* = 9 capillaries from 4 Wt mice and 13 capillaries from 5 Tg mice; *t*_*7*_ = 2.65, *F*_*1,7*_ = 7.03). Data were analyzed using nested *t* tests and are presented as biological means ± SEM. Male and female mice within each group are indicated by ο and Δ, respectively.

To attempt to bridge the disconnect between our *in vivo* and *ex vivo* data, we hypothesized that because thin-strand pericytes feed hyperpolarizing signals to cECs via gap junctions (Krolak et al., 2025), and thereby engage retrograde electrical signaling via K_ir_2.1 channels through the underlying capillary bed to dilate the upstream PA (Hariharan et al., 2022; Isaacs et al., 2024), early downregulation of cEC K_ir_2.1 signaling in 5xFAD mice could limit the upstream propagation of hyperpolarizing signals. To probe this possibility, we initially selectively pressure-ejected 10 μM pinacidil next to a TO-PRO-3-labeled thin-strand pericyte and measured diameter changes in the upstream, connected PA (Fig. 4 A). In 2–4-mo-old Wt mice, we measured an average PA dilation of 9.7% in response to this maneuver, whereas PAs in 5xFADs only dilated by 1.6% on average in response to pinacidil stimulation of downstream pericytes (Fig. 4, B and C). A change in pericyte numbers or altered vascular path length in Tg mice could contribute to decreased PA dilation to pinacidil stimulation. We thus measured the lengths of capillaries across branch orders 1–8 and assessed TO-PRO-3–positive pericyte numbers within our imaging volume but found no differences between 2–4-mo-old Wt and 5xFAD mice (Fig. S6). These data rule out structural changes as a contributory factor to impaired capillary-to-arteriole communication and suggest instead that breakdown of electrical signal propagation along the capillary bed could be the underlying cause.

Since cEC K_ir_2.1 channels enable the transmission of this pericyte-PA signal over long distances (Hariharan et al., 2022), and older (12–13 mo) 5xFAD mice show profound reductions in whole-cell cEC K_ir_2.1 current density (Mughal et al., 2021), we sought to ascertain whether similar disruption occurs in younger mice using conventional whole-cell patch clamp electrophysiology. In alignment with our *in vivo* pressure–ejection results indicating a loss of capillary-to-arteriole electrical transmission, 2–4-mo-old 5xFADs had significantly lower K_ir_2.1 current density compared with Wt’s (−4.5 vs −8.9 pA/pF, respectively; Fig. 4, D and E). To directly test cEC K_ir_2.1 function and retrograde propagation of signals *in vivo*, we then measured PA diameter in response to selective stimulation of second- or third-order capillaries of the ACT zone with 10 mM K^+^ (Fig. 4 F). 2–4-mo-old Wt mice showed robust PA dilations to this maneuver, whereas in Tg littermates, the PA dilation magnitude was significantly reduced (6.6 vs. 2.2% respectively; Fig. 4, G and H). This strongly suggested that early dysfunction of cEC K_ir_2.1 channels is a weak link that leads to disrupted transmission of pericyte K_ATP_ channel-mediated electrical signals from the capillary bed to the upstream PA.

### PIP_2_ restores endothelial K_ir_2.1 currents and retrograde propagation of electrical signals generated by pericyte K_ATP_ channels in young 5xFAD mice

**Figure 5.**
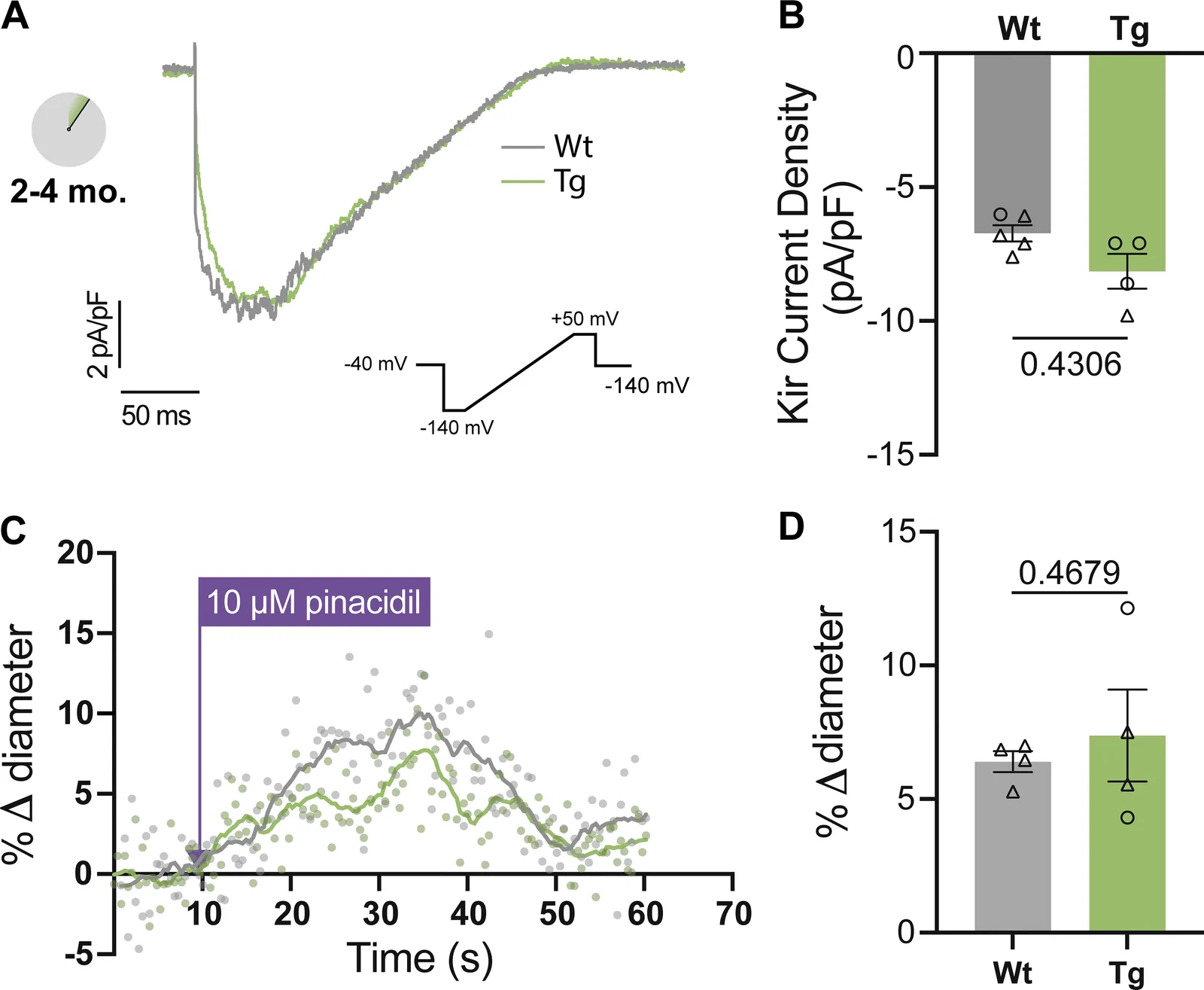
**PIP**_**2**_ **restores cEC K**_**ir**_ **2.1 currents and pericyte K**_**ATP**_ **-mediated blood flow control in 5xFAD mice. (A)** Examples of barium-sensitive K_ir_2.1 currents in freshly isolated cECs patched in the conventional whole-cell configuration with 10 μM diC_8_-PIP_2_ in the patch pipette, showing similar currents between 2–4-mo-old Wt and Tg 5xFAD mice. **(B)** Summary data showing whole-cell K_ir_2.1 currents are not significantly different between 2–4-mo-old Wt and Tg 5xFAD mice in the presence of PIP_2_ (*n* = 29 cells from 5 Wt mice and 24 cells from 4 Tg mice; *t*_*7*_ = 0.84, *F*_*1,7*_ = 0.7). **(C)** Representative traces showing similar PA diameter responses to application of pinacidil onto a fifth-order or higher pericyte between 2–4-mo-old Wt and Tg 5xFAD mice that were administered 0.5 mg/kg diC_16_-PIP_2_. **(D)** Summary of PA dilation changes to pericyte stimulation with pinacidil showing responses are not significantly different between 2–4-mo-old Wt and Tg 5xFAD mice given a systemic injection of diC_16_-PIP_2_ (0.5 mg/kg; *n* = 11 PAs from 4 Wt mice and 13 PAs from 4 Tg mice, *t*_*6*_ = 0.77, *F*_*1,6*_ = 0.6). Data were analyzed using nested *t* tests and are presented as biological means ± SEM. Male and female mice within each group are indicated by ο and Δ, respectively.

Next, we tested whether correcting cEC K_ir_2.1 dysfunction would restore capillary blood flow control by thin-strand pericyte K_ATP_ channels. Aβ peptides can interact with and decrease PIP_2_, an essential membrane phospholipid that is pivotal for K_ir_2.1 activity (Berman et al., 2008; Chauhan et al., 2000; Hansen et al., 2011; Harraz et al., 2020). Indeed, providing ECs with PIP_2_ corrects blood flow disturbances in 12–13-mo-old 5xFAD mice and mouse models of cerebral small vessel disease by increasing K_ir_2.1 current density (Dabertrand et al., 2021; Mughal et al., 2021). Therefore, we supplemented freshly isolated cECs with 10 μM diC_8_-PIP_2_ in the pipette solution and measured K_ir_2.1 currents as before. Surprisingly, even in 2–4-mo-old 5xFAD mice, this strategy significantly increased cEC K_ir_2.1 current density, restoring it to a similar level to Wt littermates (Wt: −6.7 pA/pF and Tg: −8.1 pA/pF; Fig. 5, A and B). We then tested whether this restorative effect of PIP_2_ on cEC K_ir_2.1 currents also corrects the transmission of electrical signals from pericyte K_ATP_ channels to upstream PAs in Tg 5xFAD mice. Systemic injection of 0.5 mg/kg diC_16_-PIP_2_ reinstated PA dilations to selective application of pinacidil onto a fifth-order or higher pericyte in 2–4-mo-old Tg 5xFAD mice (7.4% in Tg vs. 6.4% in Wt; Fig. 5. C and D). Overall, 2–4-mo-old Tg mice with PIP_2_ supplementation showed an ∼80% increase in cEC K_ir_2.1 current density and an approximately fivefold increase in PA dilation magnitude compared with Tg mice without exogenous PIP_2_ (Fig. 4, C and E). Collectively, these data demonstrate that a decrease in availability of the essential cofactor PIP_2_ underlies the early cEC K_ir_2.1 breakdown in 5xFAD mice and correcting this deficit restores cEC K_ir_2.1 activity and pericyte K_ATP_ channel-mediated control of blood flow.

## Discussion

The five familial AD mutations expressed by the 5xFAD mouse line produce a fast and aggressive model centered on capturing the acute effects of Aβ toxicity such as mitochondrial deficits, gliosis, neuroinflammation, synaptic loss, and cognitive decline (Oakley et al., 2006). In this study, we aimed to leverage these features to determine the consequences and timeline of familial AD mutations for blood flow control by capillary thin-strand pericytes. Our data reveal that pericyte blood flow control is lost early in the disease process in 5xFAD mice due to an initial decline in adjacent cEC K_ir_2.1 function. This severely limits the ability of hyperpolarizing signals generated by thin-strand pericyte K_ATP_ channels to reach upstream arterioles to effectively increase blood flow in response to decreases in local glucose. These findings underscore the vulnerability of the capillary bed to the primary stages of pathology in 5xFAD mice—this early and sustained loss of blood flow control may in turn exacerbate neuronal death and cognitive decline.

Along the capillary bed, pericytes exhibit complex structural, molecular, and functional heterogeneity. Unlike the dynamic and fast contractile nature of ACT zone ensheathing pericytes, deeper capillary thin-strand pericytes are less contractile (Hartmann et al., 2021) and rely on the rich complement of ion channels and G-protein–coupled receptors they express to control local blood flow via transmitted electrical signals (Hariharan et al., 2020). In these cells, message encoding the vascular K_ATP_ channel is very high, and the resultant functional channels exert a dominant influence on their membrane biology and cellular physiology (Bowen et al., 2025; Hariharan et al., 2022; Isaacs et al., 2024; Sancho et al., 2022). Indeed, activating K_ATP_ channels in thin-strand pericytes produces dilation of the upstream ACT zone capillaries and the more remote feeding PA and increases RBC flux in the capillaries from which the signal originates (Hariharan et al., 2022). Strikingly, we observed here an early and sustained impairment in vessel dilation and capillary blood flow responses to K_ATP_ channel–mediated blood flow control in 5xFAD mice. This aligns well with findings that K_ATP_-mediated dilation is affected in other AD models: 3xTgAD mice show reduced CBF responses to diazoxide at 6–8 mo, and this is completely abolished in 24–28-mo-old mice with advanced disease (Liu et al., 2021), while posterior cerebral arteries from APP mice had decreased reactivity to levcromakalim at 12 mo (Tong et al., 2012). Thus, K_ATP_ channel dysfunction may be a generalizable feature in contexts where *APP* and *PSEN1* mutations drive amyloid accumulation. The early age at onset of these defects in 5xFAD mice specifically could be due to the rapid and aggressive downstream effects of amyloid pathology in the cortex for this line (Ameen-Ali et al., 2019; Belfiore et al., 2019; Oakley et al., 2006; Whitesell et al., 2019), and the resulting early deficit in K_ATP_ function could negatively impact blood flow and oxygen delivery (Hosford et al., 2019) and thereby have a compounding effect on neuronal health and function.

### The pericyte energy switch mechanism is lost early in 5xFAD mice

Besides oxygen, brain energy production relies on a continual supply of glucose across the blood–brain barrier via GLUTs (Attwell and Laughlin, 2001; Dienel, 2019). AD is increasingly recognized as a neurometabolic disorder characterized by decreased neuronal glucose uptake and hypometabolism leading to an energy crisis that hastens neuronal decline (Johnson et al., 2020; Kyrtata et al., 2021; Mosconi, 2005; Yang et al., 2023). The energy-sensing role for thin-strand pericytes could thus be crucial in this context. In healthy conditions, K_ATP_ channels enable these cells to detect local changes in glucose availability and respond to decreases with robust corrective increases in blood flow, providing a failsafe that prevents metabolic crisis. Loss of this homeostatic energy switch mechanism could thus create or exacerbate the energy gap experienced by neurons in AD. In healthy mice, we found that blocking glucose import by GLUT1 with BAY-876 engages the pericyte K_ATP_ channel electro-metabolic switch to increase blood flow (Hariharan et al., 2022), indicating that glucose sensing in thin-strand pericytes links local substrate needs to capillary blood flow regulation. In our AD model, in contrast, we observed a selective breakdown of the pericyte energy switch in young 5xFAD mice—with dominant effects on the dilatory responses of ACT zone capillaries and capillary-level blood flow—which may thus contribute to early and progressive bioenergetic failure. It is notable that the pericytes and underlying capillaries of the ACT zone are especially vulnerable to disease states: in ischemia, pericytes can dynamically constrict capillaries and “die in rigor” (Hall et al., 2014), and ensheathing pericyte-covered capillaries remain constricted up to 90 min after reperfusion and contribute to the no-reflow phenomenon (Shrouder et al., 2024; Yemisci et al., 2009). Constriction of capillaries at the site of pericyte somata, but not arterioles, was also noted in *App*^*NL-G-F*^ mice (Korte et al., 2024; Nortley et al., 2019), suggesting differences in the susceptibilities and underlying mechanisms by which Aβ affects different segments of the vasculature. Further studies are needed to characterize segment-specific vascular deficits across a range of AD models, which may reveal common failure points.

### K_ir_2.1 dysfunction short-circuits K_ATP_ function early in 5xFAD mice

Given the reduced vessel dilation and blood flow responses to *in vivo* K_ATP_ channel activation in 5xFAD mice, which we previously demonstrated are predominantly due to activation of these channels in thin-strand pericytes (Hariharan et al., 2022), we directly assessed thin-strand pericyte membrane potential responses to pinacidil and BAY-876. Unexpectedly, pericytes from 2–4-mo-old mice still responded to both agents with membrane hyperpolarization, whereas those from 6–8-mo-old mice did not, indicating that K_ATP_ channel function is initially preserved but deteriorates with age. This presented a paradox— if K_ATP_ channels are intact in thin-strand pericytes at 2–4 mo, why do they fail to drive blood flow responses *in vivo*? Our previous work demonstrated that K_ATP_ channel-mediated signal propagation from thin-strand pericytes to upstream PAs critically depends on the capillary endothelium (Hariharan et al., 2022; Isaacs et al., 2024). In this pathway, endothelial K_ir_2.1 channels—activatable by incoming membrane hyperpolarizations and extracellular K^+^ elevations—serve as a signal generator and amplifier and thus convert the capillaries into effective electrical conduits (Longden et al., 2017). However, this is also a notable point of vulnerability, and a range of models have shown that disruption of capillary K_ir_2.1 channels attenuates the spread of hyperemic hyperpolarizing signals from capillaries to arterioles (Dabertrand et al., 2021; Mughal et al., 2021; Thakore et al., 2023). Consistent with the profound loss of cEC K_ir_2.1 activity reported in 12–13-mo-old 5xFAD mice (Mughal et al., 2021), we observed reduced K_ir_2.1 activity and diminished PA dilations in response to localized K^+^ application onto ACT zone capillaries as early as 2–4 mo of age, highlighting the remarkably early onset of capillary dysfunction in this model. Accordingly, selective *in vivo* activation of thin-strand pericyte K_ATP_ channels with pinacidil—the signals generated by which are critically dependent on cEC K_ir_2.1 channels for transmission (Hariharan et al., 2022)—failed to elicit PA dilation in 5xFAD mice. These findings, combined with overall preserved vascular density and pericyte numbers, directly link the loss of *in vivo* K_ATP_-mediated vascular responses to impaired K_ir_2.1-dependent electrical signal propagation rather than intrinsic dysfunction of thin-strand pericyte K_ATP_ channels themselves in young 5xFAD mice. Extending this to implications for capillary computation, our data indicate that early endothelial K_ir_2.1 loss will not merely attenuate electrical signals but will degrade the functional processing abilities of the CCU. Because K_ir_2.1 provides the regenerative amplification that allows pericyte-generated hyperpolarizations to be summed and propagated across the network, its failure will sever the sensor (i.e., pericyte) from its output pathway (i.e., the endothelial conduit) and thus collapse the arithmetic and gain-control operations that serve to convert distributed reports of local energy status into coordinated upstream vasodynamics (Longden, 2026). The consequence will thus not be limited to the loss of one vessel’s response but could extend to network-level computational failure: reports of metabolic need will be generated but no longer be integrated or transmitted, uncoupling substrate demand from delivery and creating a sustained energy gap that would be expected to compound neuronal dysfunction and accelerate pathology.

### PIP_2_ corrects cEC K_ir_2.1-mediated capillary-to-arteriole retrograde signaling in 5xFAD mice

PIP_2_ resides in the inner leaflet of the plasma membrane and contributes to a plethora of signaling pathways by formation of the critical secondary messengers inositol 1,4,5-trisphosphate and diacylglycerol, and through direct modulation of ion channels (Harraz et al., 2020; Hilgemann et al., 2001). PIP_2_ is a strong activator of K_ir_2.1 channels—by binding to a conserved site between the transmembrane and cytoplasmic domains of K_ir_2.1, PIP_2_ initiates a large conformational change and pore opening (Hansen et al., 2011), resulting in sustained K_ir_2.1 currents and preventing channel rundown (Harraz et al., 2018a; Huang et al., 1998). Consequently, a reduction in PIP_2_ bioavailability—as demonstrated in multiple studies relevant to AD (Berman et al., 2008; Chauhan et al., 2000; Landman et al., 2006; Stokes and Hawthorne, 1987; Zhu et al., 2015)—would be expected to profoundly impair K_ir_2.1 function.

Our data implicate PIP_2_ deficiency as the underlying cause of early K_ir_2.1 dysfunction in 2–4-mo-old 5xFAD mice. Exogenous PIP_2_ supplementation restored K_ir_2.1 current density in cECs of young 5xFAD mice and, critically, reinstated retrograde conduction of hyperpolarizing electrical signals from thin-strand pericyte K_ATP_ channels to upstream PAs. Several mechanisms could account for reduced PIP_2_ availability in this model. One plausible factor is an increase in G_q_PCR signaling mechanisms driving phospholipase C-mediated hydrolysis of PIP_2_ (Harraz et al., 2018a). For instance, levels of pro-inflammatory prostaglandin E_2_ (PGE_2_)-synthesizing enzymes are elevated in human AD brain tissue (Chaudhry et al., 2008), which might enhance PGE_2_-stimulation of G_q_-coupled EP_1_ receptors on cECs. Similarly, extracellular ATP released as an inflammatory signal from distressed cells, including hippocampal synaptosomes and microglia exposed to Aβ (Gonçalves et al., 2019; Kim et al., 2007; Sanz et al., 2009), could activate G_q_-coupled purinergic receptors in cECs and deplete the membrane PIP_2_ pool. Downstream of G_q_PCR activation, activation of cation-permeable TRPV4 or Piezo1 channels can depolarize cECs, opposing K_ir_2.1 activity and reducing blood flow (Harraz et al., 2018b; Hashad et al., 2025). Alternatively, a decrease in intracellular ATP due to mitochondrial metabolic dysfunction in AD can limit PIP_2_ synthesis and directly attenuate K_ir_2.1 current density (Alves et al., 2025; Harraz et al., 2018a; Zhang et al., 2015). Finally, decreases in Arf6—a small GTPase recently identified as an essential cofactor for maintaining physiological PIP_2_ levels in capillary ECs—could also underlie K_ir_2.1 impairment in young 5xFAD mice (Noterman-Soulinthavong et al., 2026). Collectively, these converging pathways implicate PIP_2_ deficiency as a mechanistic hub linking early Aβ-driven pathology to the loss of capillary EC blood flow control and, by extension, to overlying pericyte electrical signaling mechanisms that depend on this transmission pathway.

### Direct K_ATP_ dysfunction emerges later in 5xFAD mice

By 6–8 mo, K_ATP_ channel function itself in thin-strand pericytes had broken down in 5xFAD mice. This indicates that at more advanced stages of disease, further corrective actions may be necessary to fully revive vascular function. Here, not only would rescue of K_ir_2.1 channels be necessary but also restoration of K_ATP_ channels. Notably, the vascular isoform of these channels (i.e., K_ir_6.1, SUR2) has such high affinity for PIP_2_ that they are typically unaffected by fluctuations induced by physiological signaling events (Hariharan et al., 2020; Harraz et al., 2020). Whether this is the case in the low PIP_2_ environment of AD (Mughal et al., 2021) is unknown, but if PIP_2_ restoration is unable to preserve or correct this issue, then other approaches may be needed, such as correction of any metabolic deficits specific to pericytes, or gene therapy to restore lost channels to the membrane. Further work is thus needed to establish the mechanisms by which K_ATP_ channel dysfunction emerges in this model.

### Conclusion

Ion channels important for dynamic control of brain blood flow are impacted in AD mouse models or by acute exposure to Aβ peptides (Lim et al., 2025; Taylor et al., 2026). Using an amyloid-centric mouse model of AD, we demonstrate that capillary K_ir_2.1 endothelial dysfunction emerges early and drives a marked loss of pericyte-mediated K_ATP_ channel signaling and blood flow regulation, which can be reversed by PIP_2_ supplementation. An analogy can be drawn with demyelinating diseases, which strip nodes of Ranvier of the ability to transmit signals along axons: the early loss of K_ir_2.1 to aid transmission through the capillaries nullifies the ability of thin-strand pericytes to generate electrical signals of sufficient magnitude to reach upstream contractile elements, and thus blood flow control is compromised. Notably, these vascular deficits develop in parallel with amyloid pathology and underscore the need for therapeutic strategies aimed at preserving endothelial K_ir_2.1 function, such as restoration of PIP_2_ levels, to be implemented well before the emergence of canonical disease hallmarks such as memory impairment. As disease progresses, this early vascular impairment likely initiates a self-reinforcing cycle in which compromised capillary blood flow undermines neuronal health, while blood–brain barrier breakdown further accelerates Aβ accumulation. Together, our findings position microvascular dysfunction as an early and central contributor to AD pathogenesis and underscore the critical importance of early vascular assessment and intervention to preserve cerebral function and slow disease progression.

### Limitations

The 5xFAD line is an aggressive model of familial AD, widely used to study acute effects of amyloid accumulation that occur far more rapidly than in the human disease. Moreover, female 5xFAD mice generally exhibit worse amyloid pathology than males (Forner et al., 2021; Maarouf et al., 2013; Oakley et al., 2006; Poon et al., 2023; Sil et al., 2022). Further work should thus validate these findings in other AD models and in studies assessing factors that contribute to sporadic AD cases. Although the primary focus of this study was to determine the age of onset of capillary pericyte K_ATP_ dysfunction, future work is also needed to specifically address potential sex-dependent differences in these alterations. Beyond thin-strand pericytes, K_ATP_ channels have been identified in SMCs, ECs, and ensheathing pericytes (Sancho et al., 2022; Zambach et al., 2021). K_ATP_ channels in these cell types were not extensively examined here but may contribute to the impaired regulation of cerebral blood flow observed in 5xFAD mice. Vascular K_ir_2.1 and K_ATP_ channels play an important role in mediating functional hyperemia (Isaacs et al., 2024; Longden et al., 2017), and whether the ion channel dysfunction seen here also affects neurovascular coupling in 5xFAD mice is currently unknown. Given the overt changes in sensory integration, cortical plasticity, and early intraneuronal amyloid accumulation (Chen et al., 2022; Flanigan et al., 2014; Grant et al., 2020; Maatuf et al., 2016; Oakley et al., 2006), experiments aimed at tandem measurements of evoked neuronal activity and vascular responses are necessary to fully elucidate neurovascular coupling changes in 5xFAD mice.

## Data Availability

The data used to generate all main and supplementary figures are openly available online at Harvard Dataverse at https://doi.org/10.7910/DVN/S6WG8V.
